# Determination of steady-state transcriptome modifications associated with repeated homotypic stress in the rat rostral posterior hypothalamic region

**DOI:** 10.3389/fnins.2023.1173699

**Published:** 2023-06-09

**Authors:** Serge Campeau, Connor McNulty, Jacob T. Stanley, Anthony N. Gerber, Sarah K. Sasse, Robin D. Dowell

**Affiliations:** ^1^Department of Psychology and Neuroscience, University of Colorado, Boulder, CO, United States; ^2^Molecular, Cellular and Developmental Biology, University of Colorado, Boulder, CO, United States; ^3^BioFrontiers Institute, University of Colorado, Boulder, CO, United States; ^4^Department of Medicine, National Jewish Health, Denver, CO, United States; ^5^Department of Medicine, University of Colorado, Aurora, CO, United States; ^6^Department of Computer Science, University of Colorado, Boulder, CO, United States

**Keywords:** habituation, RNA-seq, sound, glucocorticoids, chronic stress, homotypic, gene regulation

## Abstract

Chronic stress is epidemiologically correlated with physical and psychiatric disorders. Whereas many animal models of chronic stress induce symptoms of psychopathology, repeated homotypic stressors to moderate intensity stimuli typically reduce stress-related responses with fewer, if any, pathological symptoms. Recent results indicate that the rostral posterior hypothalamic (rPH) region is a significant component of the brain circuitry underlying response reductions (habituation) associated with repeated homotypic stress. To test whether posterior hypothalamic transcriptional regulation associates with the neuroendocrine modifications induced by repeated homotypic stress, RNA-seq was performed in the rPH dissected from adult male rats that experienced either no stress, 1, 3, or 7 stressful loud noise exposures. Plasma samples displayed reliable increases of corticosterone in all stressed groups, with the smallest increase in the group exposed to 7 loud noises, indicating significant habituation compared to the other stressed groups. While few or no differentially expressed genes were detected 24-h after one or three loud noise exposures, relatively large numbers of transcripts were differentially expressed between the group exposed to 7 loud noises when compared to the control or 3-stress groups, respectively, which correlated with the corticosterone response habituation observed. Gene ontology analyses indicated multiple significant functional terms related to neuron differentiation, neural membrane potential, pre- and post-synaptic elements, chemical synaptic transmission, vesicles, axon guidance and projection, glutamatergic and GABAergic neurotransmission. Some of the differentially expressed genes (Myt1l, Zmat4, Dlx6, Csrnp3) encode transcription factors that were independently predicted by transcription factor enrichment analysis to target other differentially regulated genes in this study. A similar experiment employing *in situ* hybridization histochemical analysis in additional animals validated the direction of change of the 5 transcripts investigated (Camk4, Gabrb2, Gad1, Grin2a and Slc32a) with a high level of temporal and regional specificity for the rPH. In aggregate, the results suggest that distinct patterns of gene regulation are obtained in response to a repeated homotypic stress regimen; they also point to a significant reorganization of the rPH region that may critically contribute to the phenotypic modifications associated with repeated homotypic stress habituation.

## Introduction

Despite its imprecise definition, stress is epidemiologically associated with physical and psychiatric disorders (Kessler, 1997; Kendler et al., 1999; Segerstrom and Miller, 2004). Psychopathologies are frequently correlated with repeated or chronic exposure to stressors (Pasternac and Talajic, 1991; Chrousos and Gold, 1992; McEwen, 1998, 2000, 2004; Chrousos, 2000; Morrison, 2001), which over time, is believed to facilitate or sensitize stress responses (Morris et al., 2010; Herman, 2013; Ursin, 2014; Belda et al., 2015; Post, 2016). Several animal paradigms of chronic intermittent stress also sensitize stress-associated responses and model some human pathological symptoms (Ortiz et al., 1996; Willner, 1997; Bielajew et al., 2002; Grippo et al., 2005; Ostrander et al., 2006; Bondi et al., 2008; Hill et al., 2010; Cox et al., 2011; Belda et al., 2015). However, not all repeated stress regimens lead to response sensitization, but instead produce response reductions defined as habituation, especially when the recurrent intermittent threat is moderate and nearly always the same or homotypic (Grissom and Bhatnagar, 2009; Rabasa et al., 2015; McCarty, 2016; Hughes B. M. et al., 2018; Radley and Herman, 2022). While the characteristics that predictably lead to response sensitization or habituation are not fully understood and may even occur concurrently across different responses (Ottenweller et al., 1989; Hauger et al., 1990; Bhatnagar and Dallman, 1998), several established paradigms consistently induce these divergent adaptive responses. Surprisingly, whereas response sensitization has been emphasized as an important etiological factor in stress-related disorders, stress habituation has been mostly ignored despite a strong association between impaired stress habituation and multiple mood and anxiety disorders (Malmo et al., 1951; Lader and Wing, 1964; Koepke and Pribram, 1967; McGuinness, 1973; Brierley and Jamieson, 1974; Raskin, 1975; Chattopadhyay et al., 1980; Reus et al., 1985; Metzger et al., 1999; Rothbaum et al., 2001; Campbell et al., 2014). Furthermore, individuals with personality types predictive of high incidences of cardiovascular disease regularly exhibit impaired stress response habituation (Johnson et al., 2012; Howard and Hughes, 2013; Hughes B. M. et al., 2018). These observations suggest that *failure to reduce* stress-related responses upon experiencing routine challenges may itself contribute to the cumulative adverse consequences of repeated stress.

Habituation to repeated homotypic stress employing different moderate stimulus modalities reduce responses in multiple effector domains (Watts, 1975; Zbrozyna, 1976; Abbott et al., 1984; Abercrombie and Jacobs, 1987; McCarty et al., 1992; Dobrakovova et al., 1993; Chen and Herbert, 1995; Yu and Blessing, 1997; Marti and Armario, 1998; Campeau et al., 2002; Carter et al., 2004; Grissom et al., 2008; Masini et al., 2008, 2011). The rostral posterior hypothalamus (rPH) is one of the few brain regions that controls multiple stress-related responses (DiMicco et al., 2002; Kataoka et al., 2014; Myers et al., 2016; Nyhuis et al., 2016). Importantly, silencing of the rPH region with muscimol not only impairs neuroendocrine, cardiovascular, and hyperthermic acute reactions to different stress situations (Stotz-Potter et al., 1996; Morin et al., 2001; Myers et al., 2016; Nyhuis et al., 2016), it also diminishes the acquisition of neuroendocrine habituation to at least two different repeated stressors (Nyhuis et al., 2016). The molecular underpinnings of rPH modifications responsible for these habituated responses are currently unknown. Several studies have investigated transcriptional regulation in the context of repeated stress (Surget et al., 2009; Sasse et al., 2013; Gray et al., 2014; Rubin et al., 2014; Ding et al., 2015; Malki et al., 2015; Bagot et al., 2016, 2017). Some of these studies (Gray et al., 2014; Bagot et al., 2016, 2017) were effective in identifying new transcriptional profiles that have begun to define novel adaptive molecular pathways in different brain regions. However, these determinations have not been carried out with habituation-inducing repeated stress protocols that include examination of posterior hypothalamic regions. The present study was therefore designed to assess whether a repeated homotypic stress regimen associated with neuroendocrine habituation induces a measurable transcriptional response in the rPH region. The habituation model employed repeated exposures to loud noises, which induce sizable acute neuroendocrine, autonomic, and behavioral responses (Henkin and Knigge, 1963; Borrell et al., 1980; Britton et al., 1992; Campeau and Watson, 1997; Masini et al., 2008, 2011). Importantly, the large HPA axis, heart rate, core body temperature, and locomotor activation (escape) responses triggered by an initial loud noise all display significant habituation after repeated loud noise presentations (Armario et al., 1984; Masini et al., 2008, 2011; Bourin, 2015). The initial RNA-seq results suggested a time-dependent transcriptional response that tracked the amplitude of habituated neuroendocrine responses at the rPH level. Some of these transcriptional patterns were confirmed employing *in situ* hybridization histochemical techniques in an independent study, which further assessed and supported temporal and spatial specificity of transcriptomic modifications to the rPH region.

## Methods

### Animals

Young adult male Sprague–Dawley (SD) rats (300–325 g) were obtained from Envigo and housed into clear plastic tubs with food and water constantly available. The decision to carry out these initial studies in males was based on evidence available in SD males only (rPH role in stress habituation; Nyhuis et al., 2016). Conditions in the rat colony were controlled to constant humidity and temperature, with a 12:12 h light/dark cycle (lights on at 7:00 am). Testing was performed between 8:30 am and 12:30 pm during the circadian nadir of HPA axis activity. All procedures were reviewed and approved by the Institutional Animal Care and Use Committee of the University of Colorado Boulder and conformed to the United States of America National Institute of Health Guide for the Care and Use of Laboratory Animals. All efforts were made to minimize animal suffering and the number of animals used.

### RNA-seq study

#### Experimental design

For the initial RNA-seq study, 24 rats were handled and transported to an experimental room and placed daily into sound-attenuating chambers (Masini et al., 2008) for 7 consecutive days. They were randomly assigned to 4 different groups (*n* = 6/group) exposed to either 1 (AS – Acute Stress), 3 (RS3 – Repeated Stress 3), or 7 (RS7 – Repeated Stress 7) loud noises [30-min white noise at 100 decibels, A scale [dBA] sound pressure level [SPL] once/day (Masini et al., 2008)], or a home-cage background noise (fan noise ~55 dBA SPL) control (C) group. Only rats experiencing 7 loud noise exposures were expected to display significant corticosterone reduction (habituation) compared to the acute noise exposed group (Masini et al., 2008). The daily noise schedule was organized such that the last noise or control exposure occurred on day 7 for all rats (see Table 1), when they were sampled for blood (~0.3 μl via a small lateral vein tail nick under gentle restraint, within 2-min) immediately at the end of the noise or control exposures. Whole blood was spun to obtain plasma which was assayed with a commercial kit (Arbor Assays #K014-H1) according to the manufacturer’s protocol, to determine individual corticosterone (CORT) levels. All rats were euthanized on the same morning, with rats from each of the four groups euthanized sequentially, 24 h after the last noise (or control) exposure, to minimize putative transcriptional effects associated with acute stress/blood sampling or time of day/order of euthanasia. For euthanasia, rats were transported in their home cages to a quiet holding room outside of the housing colony, where they were kept for 2 h, until euthanasia was performed in an adjacent, but different, room via guillotine after minimal disturbances to the remaining rats in their home cages. Within 2 min from retrieving each rat from its cage, brains were rapidly extracted and placed in an ice-cold large rat brain matrix (EMS #69083-C – coronal, 1.0 mm), and a 2-mm coronal brain slice between 2.5–4.5 mm posterior to bregma (including the rPH region bilaterally; Paxinos and Watson, 2004) was collected and submerged in 2 mL of RNA*later* RNA Stabilization Solution (Invitrogen) at 4°C. The brain slices were further dissected 24-h after collection on an ice-cold surface by excising a block of tissue bounded ventrally by a horizontal cut 0.5-mm ventral to the dorsal aspect of the 3^rd^ ventricle, laterally by vertical cuts centered on the lateral edge of the mammillothalamic tracts, and dorsally by a horizontal cut centered on the dorsal edge of the mammillothalamic tracts; the tissue block was submerged in RNALater and stored at 4°C until RNA extraction (6 days).

**Table 1:** RNA-seq experimental design.

C	–	–	–	–	–	–	–: bs	Eut
AS	–	–	–	–	–	–	x: bs	Eut
RS3	–	–	–	–	x	x	x: bs	Eut
RS7	x	x	x	x	x	x	x: bs	Eut
Days	1	2	3	4	5	6	7	8

Groups of rats (*n* = 6/group) were exposed to 30 min of white noise (x), 100 decibels, A scale (dBA) sound pressure level (SPL), or no noise (−) on consecutive days. Blood samples (bs) were collected immediately after exposure on day 7 and assayed for corticosterone (CORT). Animals were euthanized (Eut) 24 h after the last noise or no noise exposure for brain tissue collection and RNA extraction. C (control): no noise control group; AS (acute stress): single loud noise exposure; RS3 (repeated stress 3): three loud noise exposures; RS7 (repeated stress 7): seven loud noise exposures.

#### Library preparation, sequencing and data analysis

Total RNA was extracted (PureLink RNA Mini Kit #12183018A with TRIzol Reagent #15596026, Invitrogen) and quality assessed with RNA Integrity (RIN) scores determined by an Agilent 2100 Bioanalyzer. RNA quality was very high across all animals, with average RIN scores of 8.64 (st.dev.: 0.28 – range: 7.9–9.1). Uniquely indexed, paired-end (75 bp) libraries were prepared with a KAPA HyperPrep mRNA kit (#KK8580) and sequenced on an Illumina NextSeq 500 sequencer (Biofrontiers Institute, University of Colorado Boulder). RNA library sequencing resulted in low amplifications in four libraries/rats. Raw reads were examined for quality using FastQC (v. 0.11.5); this quality check indicated that the lowest quality libraries had either the fewest reads (1 rat: 8.29 million reads) or had excessive overrepresentations of adapter sequences and high GC content (3 rats), so these rats, one in each of the experimental conditions, were excluded from further analyses. Sequences were trimmed for quality, length and adapter contamination using Trimmomatic (v. 0.36), and mapped to the rn6 *Rattus Norvegicus* reference genome (downloaded June 2020^1^) using HISAT2 (v. 2.0.5). In the 20 rats retained for differential expression analysis, the average number of reads was 20.44 million reads per library/rat (standard deviation: 3.788; range: 13.05–26.99 million reads). Gene expression was estimated using the featureCount routine of the Subread module (v. 1.6.2) on sorted BAM files with the annotated rat genome (Rnor 6.0.100). These gene count estimations were then used independently as DESeq2 inputs (v. 1.28.1 (Love et al., 2014); run in R v. 4.0.2) for differential gene expression analyses to report significant log2 fold changes (lfc) with *p_adj_* values (Wald statistic) of ≤0.05. Gene ontology analyses, compared to the control condition and between stress conditions, were performed in DAVID^2^ (Huang et al., 2009; Sherman et al., 2022). Transcription factor enrichment analyses were performed with ChEA3^3^ (Keenan et al., 2019). These analyses prioritize candidate regulatory transcription factors based on the overlap between given lists of differentially expressed genes, and previously annotated transcription factor targets assembled from multiple sources (ENCODE, ReMap, GTEx, ARCHS4, Enrichr, and gene signatures resulting from single transcription factor perturbations followed by genome-wide gene expression experiments). The top 10 ranking transcription factors from the integrated mean rank libraries in ChEA3 are reported, as this method consistently performed better in benchmark tests of large numbers of ChIP-seq and RNA-seq studies (Keenan et al., 2019).

### Validation study with *in situ* hybridization histochemistry

#### Experimental design

This study assessed 5 target genes that were identified as significantly regulated by repeated stress habituation in the RNA-seq study and focused on genes that: (1) mediate neural communications (synaptic transmission-associated proteins); or (2) contribute to neural plasticity. As an independent test to assess the validity of this transcriptomic regulation, 30 additional rats were treated exactly as described for the RNA-seq study above, with the following modifications. Rats were randomly assigned to 5 different conditions, including exposure to either 1 (*n* = 12), 3 (*n* = 6), or 7 (*n* = 6) loud noises, or a home-cage background noise (*n* = 6) control group. Rats exposed to one loud noise were divided into two conditions, with one group exposed to loud noise 24-h prior to euthanasia (same condition as in RNA-seq study; *n* = 6), and a second group exposed to loud noise 8 days prior to euthanasia (*n* = 6), as shown in Table 2. This additional condition was included to test the possibility that the larger transcriptomic response observed in the 7-loud noise exposed group of the initial study is simply the result of a delayed transcriptomic response to even a single stress exposure experienced 8 days earlier. All rats were again sampled for blood as described in the RNA-seq study immediately at the end of day 7’s noise or control exposures for the determination of individual CORT levels. All rats were euthanized on the same morning, with rats from each of the five groups euthanized sequentially, 24 h after the last noise (or control) exposure as described in the RNA-seq study. Brains were rapidly extracted, frozen in -30°C isopentane, and stored at −80°C until sectioned (10 μm sections) in a cryostat (Leica 1850, Leica Microsystems, Buffalo Grove, IL, United States). Coronal sections were obtained from each rats’ brain in 3 different regions including the central amygdaloid nucleus (ACe, 1.9–2.4 mm posterior to bregma), the posterior hypothalamic region (rPH, 3.2–3.4 mm posterior to bregma) and the closely located dorsomedial hypothalamic nucleus (DMH, 2.9–3.2 mm posterior to bregma; Paxinos and Watson, 2004) to assess the regional specificity of habituation-related transcript regulation. The transcripts selected from the RNA-seq study included Gabrb2, Grin2a, Camk4 (upregulated genes), Gad1 and Vgat (downregulated genes), due to the possibility that these genes may significantly contribute to rPH plasticity in response to repeated homotypic stress exposures and availability of robust and specific sense and antisense complementary RNA (cRNA) probes (see Table 3). Sections were thaw-mounted onto polylysine-coated glass slides, quickly frozen, and stored at −80°C until assayed by radiometric *in situ* hybridization (ISH) against target mRNAs of interest.

**Table 2:** Validation (*in situ* hybridization histochemistry) experimental design.

C	–	–	–	–	–	–	–: bs	Eut
AS7	x	–	–	–	–	–	–: bs	Eut
AS1	–	–	–	–	–	–	x: bs	Eut
RS3	–	–	–	–	x	x	x: bs	Eut
RS7	x	x	x	x	x	x	x: bs	Eut
Days	1	2	3	4	5	6	7	8

Groups of rats (*n* = 6/group) were exposed to 30 min of white noise (x), 100 dBA SPL, or no noise (−) on consecutive days. Blood samples (bs) were collected immediately after exposure on day 7 and assayed for CORT. Animals were euthanized (Eut) 24 h after the last noise or no noise exposure for brain collection. C (control): no noise control group; AS1/7: single loud noise exposure 1 or 7 days prior to euthanasia; RS3: three loud noise exposures; RS7: seven loud noise exposures.

**Table 3:** *In situ* hybridization histochemistry complementary RNA (cRNA) probe details.

Camk4: Reference sequence NM_012727.3; 688 bp fragment (256. … 943) spanning exons 4–14.
Gabrb2: Reference sequence NM_012957.2; 688 bp fragment (81. … 768) spanning exons 2–7.
Gad1: Reference sequence NM_017007; 210 bp fragment (1836. … 2046) within the last exon (gifted from Drs. Stanley Watson and Huda Akil, University of Michigan).
Grin2a: Reference sequence NM_012573.3; 688 bp fragment (1,114. … 1801) spanning exons 4–8.
Slc32a: Reference sequence NM_031782.1; 688 bp fragment (101 … 788) spanning exons 1 and 2.

#### Radiometric ISH histochemistry

The methods for radiometric ISH were similar to those described previously (Babb et al., 2013; Sasse et al., 2013). Briefly, slides were fixed, treated to reduce non-specific cRNA binding, and hybridized with ^35^S-UTP-labeled cRNA probes overnight, washed and exposed to X-ray films for 6–10 days. Test films were included with slides run concurrently and were used to optimize the exposure time of the films containing experimental slides. Levels of mRNA from films were analyzed by computer-assisted optical densitometry by an experimenter blind to the treatment conditions. Images of each individual brain section were captured digitally (CCD camera, model XC-77; Sony, Toyko, Japan), and analyzed using Scion Image (Version 4.03 for Windows; ScionCorp). Prior to analysis, signal for all groups was verified to be within the linear range, with all pixels being contained within 30–200 (of a 1–250 max range) to ensure that hybridization was not saturated. The relative optical density of the x-ray film was determined using a macro within Scion Image (written by S. Campeau) which allowed the automatic determination of a signal above background. Specifically, for each section, a background sample was taken over an area of white matter, and the signal threshold was set as 3.5 standard deviations above the mean gray value of the background. The remaining pixels above this threshold were then analyzed within the region of interest. For consistency, a different template was created for each brain region, and was placed using anatomical landmarks based on the white matter distribution of the unstained tissue, according to a standard rat brain atlas (Paxinos and Watson, 2004). The number of pixels above background was multiplied by the signal above background to give an integrated density value for both hemispheres throughout the rostral-caudal extent of each brain region of interest. The mean integrated densities for each animal were then calculated by averaging the integrated density values from 1 to 2 brain sections bilaterally (a total of 2 to 4 values), resulting in a single value for each animal representing the relative mRNA expression for each brain region of interest. These raw values were used for statistical analyses (see below). Log2 fold changes were then calculated for each cRNA and brain region to allow for the display of multiple brain regions with very different levels of mRNA expression on the same graph, and to provide relative comparisons to the RNA-seq results.

### Statistical analyses

All CORT values were analyzed with appropriate one-way ANOVAs, with significance set at *p* = 0.05. Further *post-hoc* mean comparisons were performed using Bonferroni’s corrections. Raw values of ISH mRNA expression integrated densities were analyzed using pre-planned contrasts between the control (C) and every other group (AS7, AS1, RS3 and RS7) within the general linear model implemented in R Statistical package (version 4.0.0 for Windows), with statistical significance for these tests set at *p* = 0.01 to correct for the relatively large number of contrasts performed. The built-in dummy contrast method was used for the contrasts in each brain region and transcript measured. For presentation purposes only, raw integrated densities results were transformed into Log2 fold changes against the appropriate control groups for a rough comparison to the RNA-seq results. Pearson’s product–moment correlation coefficients and associated statistics (*p* ≤ 0.05) were computed between the CORT values and the respective mean integrated densities for rats that were exposed to stress 24-h prior to euthanasia (AS1, RS3 and RS7) in the *in situ* experiment to determine if significant correlations exit between CORT and the 5 mRNAs in the 3 brain regions examined.

## Results

### RNA-seq study

**Figure 1:**
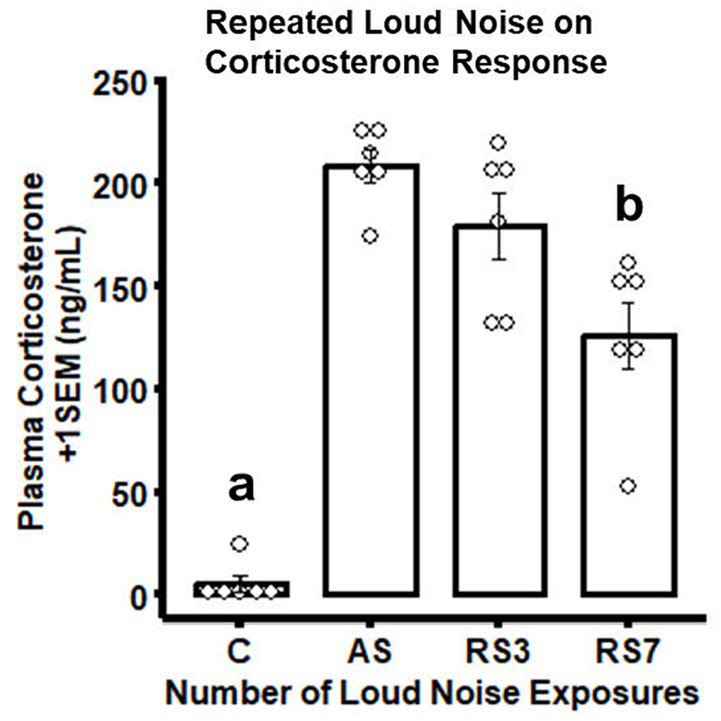
Combined bar/dotplots illustrating individual plasma corticosterone levels (ng/ml – white dots), group means (bars) and standard errors of the mean (SEM) achieved in the initial RNA-seq study in the control (C) and loud noise exposed rats [1(AS), 3(RS3) and 7(RS7) sessions of 30 min loud noise (100 dBA SPL; *n*=6/group)]. ^a^Denotes significant differences between the C group and all other groups (*post-hoc* comparisons with Bonferroni correction, *p* ≤ 0.05); ^b^Indicates significant differences between the RS7 and the AS and RS3 groups, respectively (*post-hoc* comparisons with Bonferroni correction, *p* ≤ 0.05).

As expected (Masini et al., 2008), rats experiencing the largest number of loud noise exposures (RS7) displayed a significant reduction in CORT levels on the last stress day, as compared to the groups exposed to 1 or 3 loud noises, indicating reliable habituation (Figure 1). The control (C - no noise) group had the lowest CORT levels, while a single loud noise exposure (AS) was associated with the largest CORT response. Seven loud noise exposures reliably reduced CORT levels at the end of the 30 min noise exposure compared to the 1 and 3 loud noise-exposed groups. These findings were supported by a one-way ANOVA, indicating a significant effect of differential loud noise exposures (*F*_3,20_ = 48.05, *p* < 0.05). *Post-hoc* mean comparisons (Bonferroni correction) indicated that while the control group significantly differed from the other groups (*p*’s < 0.05), the RS7 group differed significantly from the AS and RS3 groups (*p*’s < 0.05), but the latter groups did not differ from each other (*p* > 0.05).

**Figure 2:**
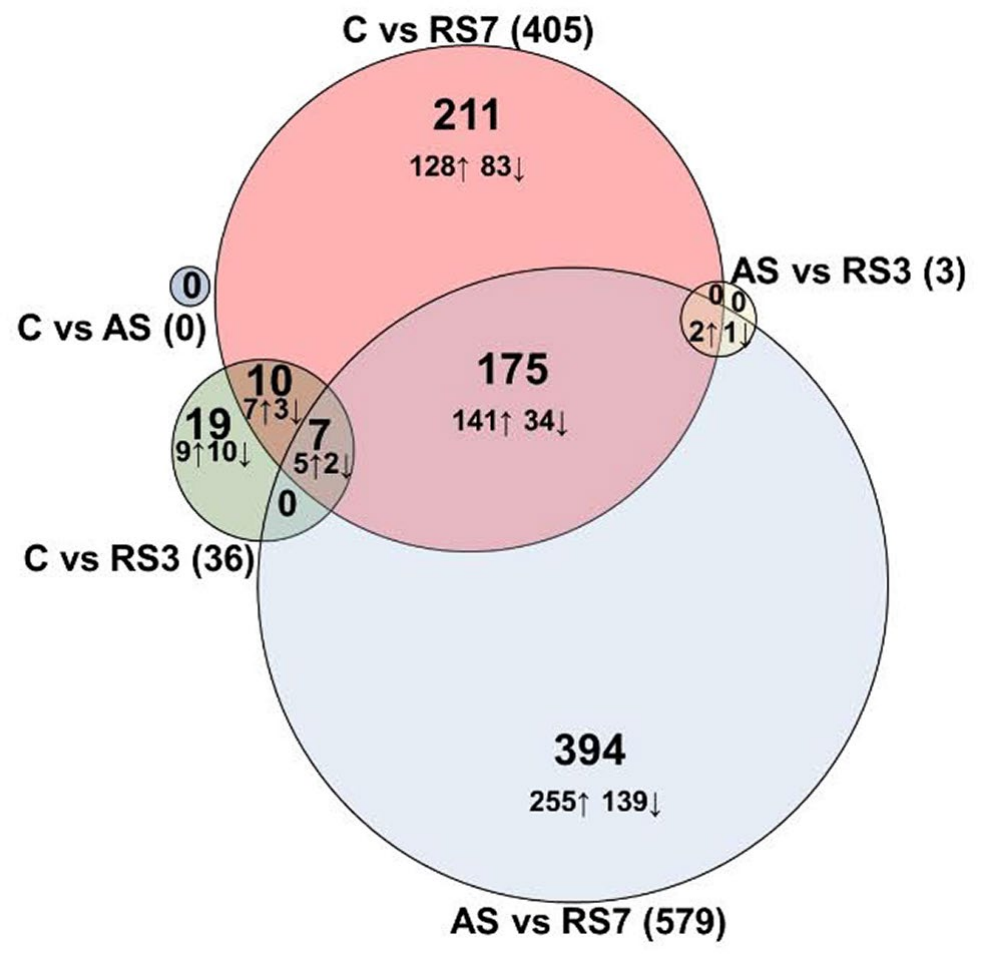
DESeq2 differentially expressed genes. Differential expression analyses of mRNA obtained 24 h after the last control or stress exposure. The number of genes that displayed significant log2 fold changes increase (↑) or decrease (↓) in rats exposed to 1 (AS), 3 (RS3) or 7 (RS7) loud noises, respectively, compared to the no noise control group and between the different stress groups (Ward statistic, *p_adj._* ≤ 0.05). Log2 fold changes varied from −4.18 to 1.65, with the largest values observed in the AS versus RS7 comparison.

**Figure 3:**
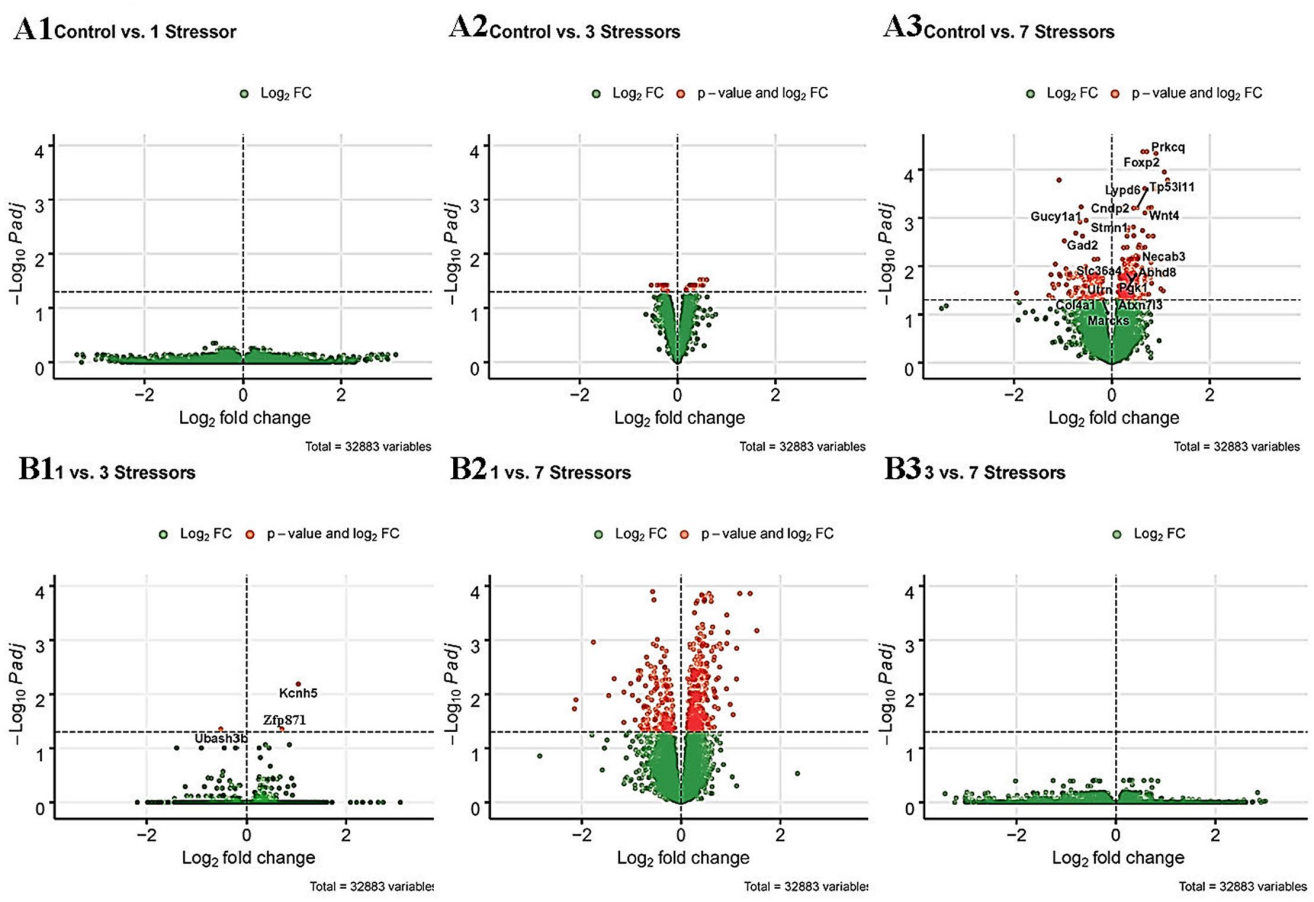
A1-3. Volcano plots (Blighe, 2018) of the log2 fold changes measured between the C versus AS (A1), C versus RS3 (A2) and C versus RS7 (A3) comparisons. The gene names presented in the Control versus 7 stressors (A3) are the 17 genes that were common with the gene list reported after the control versus 3 stressors differential expression analysis. B1-3. Volcano plots of the log2 fold changes measured between the AS versus RS3 (B1), AS versus RS7 (B2) and RS3 versus RS7 (B3) comparisons. The green dots represent all the detected transcripts that were below statistical significance (Ward statistic, *p_adj._* ≥ 0.05); the red dots illustrate the transcripts that were above significance (Ward statistic, *p_adj._* ≤ 0.05) in the respective comparisons.

Following euthanasia and tissue collection in the rPH region 24-h after the last control or loud noise exposure, RNA was extracted from tissue blocks and processed for RNA-seq. Multiple comparisons for differential gene expression were examined, including C versus AS, C versus RS3, C versus RS7, AS versus RS3, AS versus RS7 and RS3 versus RS7 groups. Genes and associated statistics are listed in Supplemental Table S1. There were no significantly altered genes (DESeq2 Wald Statistic; *p*_adj_ ≤ 0.05) in the C versus AS group comparison, as shown in Figure 2. After 3 loud noise exposures, 36 differentially expressed genes were obtained when compared to the control group; 3 of those were uncharacterized sequences. A similar analysis between the C and RS7 conditions reported 405 differentially expressed genes, as shown in Figure 2; 20 of those were poorly characterized sequences or loci. Of the 33 previously characterized genes differentially expressed in the C versus RS3 group comparison, 17 were also contained in the C versus RS7 list, but the log2 fold changes were typically larger in the RS7 comparison. Contrasts amongst the stress groups mirrored many of the results from the control comparisons. For example, there were only 3 differentially expressed genes in the AS versus RS3, and none in the RS3 versus RS7 group contrasts (Figure 2). The largest number of differentially expressed genes were observed in the AS versus RS7 group comparison, with a total of 579; a total of 184 of these differentially expressed genes were shared with the list from the C versus RS7 group comparison (405), indicating an overlap of 45% between these two contrasts. There were 7 differentially expressed genes shared between the C versus RS3, C versus RS7 and AS versus RS7 group comparisons, including Gad2, Gucy1a1, Cndp2, Foxp2, Lypd6, Prkcq and Wnt4. Plots of the log fold changes of the differential gene expression analyses from all these comparisons (volcano plots) are shown in Figure 3. These results suggest that there is a progressive effect of stress exposures and a positive relationship between the magnitude of transcription modifications and the number of stress sessions experienced prior to euthanasia; this explains the increase in the number of genes differentially expressed in the rPH region between the groups with the fewest loud noise exposures (C and AS) and the group with the larger number of stress exposures (RS7), and the relative lack of differences for intermediate comparisons.

**Figure 4:**
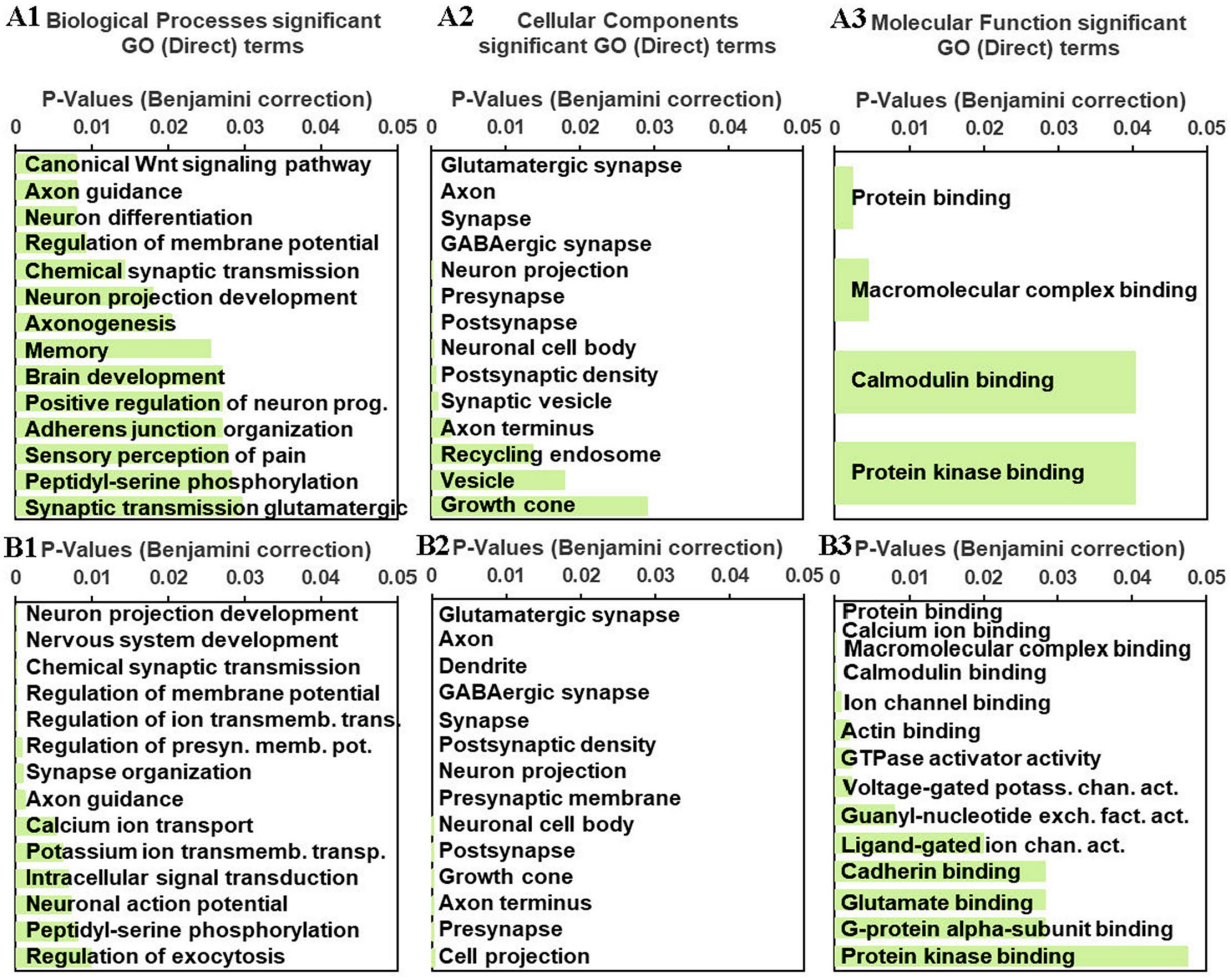
A1–3: gene enrichment/ontology (GO) analyses results for significant biological processes (A1), cellular components (A2) and molecular functions (A3) from the C versus RS7 group comparison gene list processed in DAVID (see text). B1-3. Similar gene enrichment analyses were performed on the AS versus RS7 group comparison gene list. The green horizontal bars represent the significance levels (Benjamini corrections, *p* ≤ 0.05) associated with the indicated GO terms.

To help profile meaningful patterns of transcriptomic changes induced by an habituating stress regimen in the rPH region, gene ontology (GO) analyses were performed on the gene lists obtained from the C versus RS3, C versus RS7 and AS versus RS7 comparisons. The relatively small number of genes differentially expressed in the C versus RS3 group comparison was enriched for one significant functional annotation cluster which included zinc finger/binding/nuclear annotations (Benjamini *p_adj_* = 0.023). The set of differentially expressed genes from the C versus RS7 group comparison provided multiple significant gene enrichment categories, as shown in Figure 4. The gene categories most strongly associated with repeated homotypic stress exposures included genes in the Wnt signaling pathway and genes that regulate neural differentiation, memory, membrane potential, axons, pre- and post-synaptic elements especially related to GABAergic and glutamatergic neurotransmitter systems, with reliable calcium and kinase activity associated genes. For example, multiple subunits of the GABA_A_ receptor family (Gabrb1, Gabrb2, Gabrd), the glutamate decarboxylase enzymes (Gad1, Gad2), and the vesicular GABA transporter (Slc32a1) largely contributed to the GABAergic neurotransmitter system GO term. Similar analyses from the AS versus RS7 group contrast provided relatively similar biological processes; the apparent differences were almost always produced by the terms in the AS versus RS7 list being significant but pushed below the top 10 terms (axonogenesis, memory, brain development), with the exception of the canonical Wnt signaling pathway, which was not significant. The cellular components of the AS versus RS7 list were very similar, including the ordering, of most of the significant terms in comparison to the C versus RS7 list. The molecular function terms were likewise similar; because of the larger number of differentially expressed genes in the AS versus RS7 group comparison, a greater number of terms were significant.

**Figure 5:**
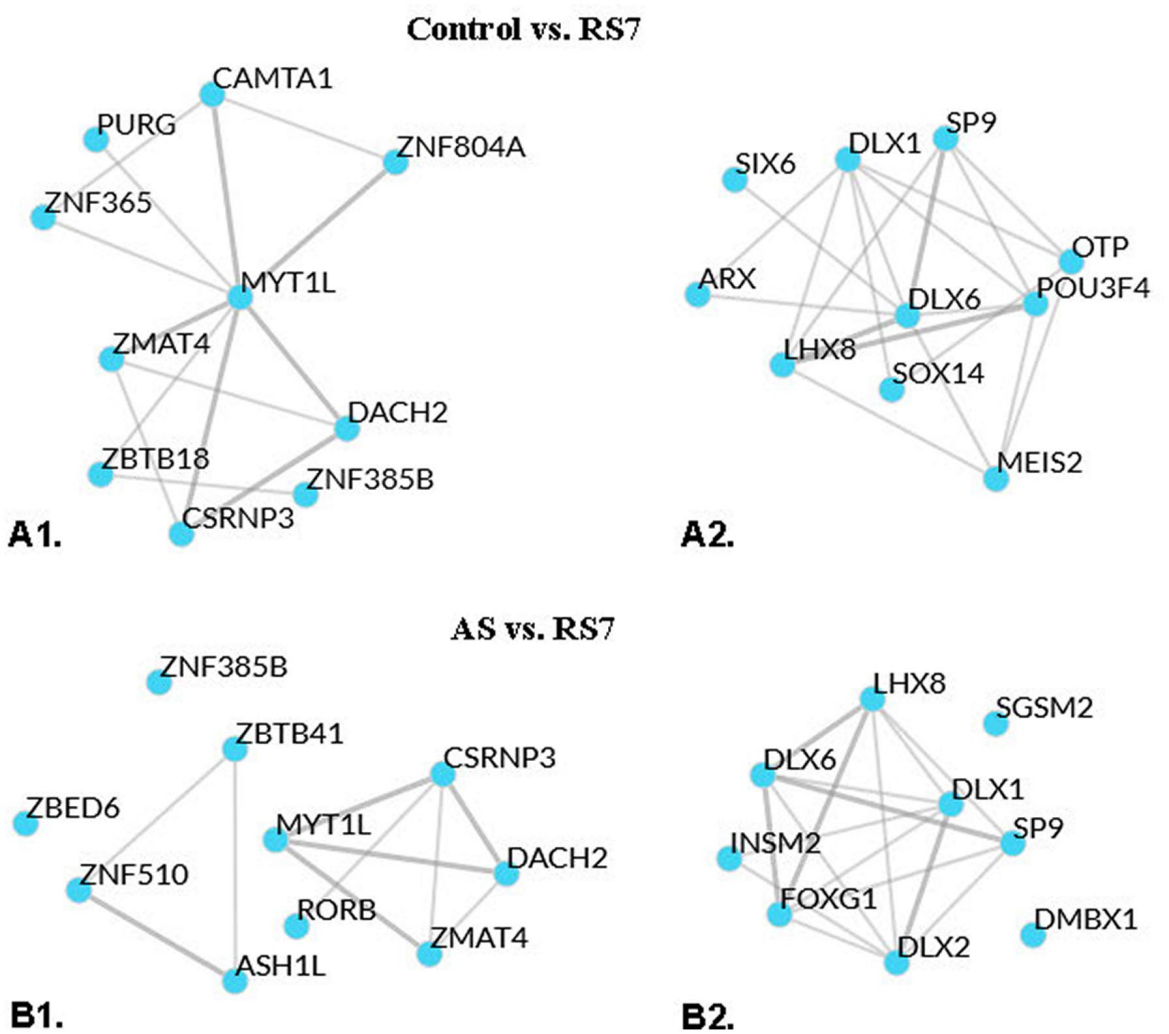
Local network analysis representation of top means ranked transcription factors displaying putative interactions, based on averaged integrated ranks across multiple libraries and databases (see ChEA3 libraries). **A1**: local network interaction results from the top 10 mean rank transcription factors of the control versus RS7 group comparison upregulated gene list. **A2**: local network interaction results from the top 10 mean rank transcription factors of the control versus RS7 group comparison downregulated gene list. **B1**: local network interaction results from the top 10 mean rank transcription factors of the AS versus RS7 group comparison upregulated gene list. **B2**: similar analysis for the AS versus RS7 group comparison downregulated gene list. Thicker lines indicate multiple library evidence for putative associations of the respective transcription factors.

A transcription factor enrichment analysis was next performed to predict regulatory factors/networks systematically enhanced or repressed by repeated homotypic stress in the rPH region based on the observed differential gene expression. Transcription factor analysis on the gene list reported for the C versus RS3 group comparison (including both up- and down-regulated genes) provided a few high-ranking transcription factor associations (top 2: Fbxl19 and Mypop, with mean ranks of 20.5 and 28.5, respectively; lower numbers imply stronger putative associations), while that for the C versus RS7 comparison provided transcription factor associations with mean ranks ranging from 11.0 (Myt1l – myelin transcription factor 1-like) to 29.0 (Dach2 – Dachshund family transcription factor 2). The top 10 transcription factors based on ChEA3 mean rank method for the C versus RS7 comparison are listed in Supplemental Table S2. Only one of those 10 transcription factors, Zmat4 (Zinc finger, matrin-type 4 - mean rank: 13.0), was a member of the differentially expressed gene list reported in the C versus RS7 group comparison. Not surprisingly, 9 of the 10 top transcription factors reporting multiple associations with the genes from the C versus RS7 comparison were highly represented in brain tissue, based on the Genotype Tissue Expression library (GTEx TF network). Similar analyses were performed separately on the C versus RS7 group comparison of reported up- and down-regulated genes, respectively, to determine if closer or different associations could be obtained (see Supplemental Table S2). For the C versus RS7 list of up-regulated genes (283), Mytl1 still came in the top mean rank (8.0), with Zmat4 (17.0) still within the top 10 mean ranked transcription factors. Putative interactions between these transcription factors are represented graphically in Figure 5A1. With the exception of one transcription factor (Peg3), the top 10 mean rank transcription factors enriched in the whole list (up- and down-regulated) were the same as in the up-regulated C versus RS7 gene list. For the C versus RS7 list of down-regulated genes (122), a different set of top mean rank transcription factors emerged (see Figure 5A2), with Dlx6 (Distal-less homeobox 6) at the top (mean rank: 9.33), which was also in the list of down-regulated genes. None of the top 10 transcription factors associated with gene down-regulation were members of the top 10 transcription factors associated with up-regulation of the C versus RS7 comparison. Of interest, many of the top mean ranked up-regulated transcription factors (Myt1l, Znf365, Camta1, Znf385b) were associated with up-regulated transcripts encoding subunits of the GABA_A_ receptor family, while nearly all top mean ranked down-regulated transcription factors were associated with down-regulated Gad1 (Gad67), Gad2 (Gad65) and Slc32a1 (Vgat) transcripts. Similar transcription factor enrichment analyses were carried out on the AS versus RS7 group comparison gene list with many of the same transcription factors displaying the strongest associations to the differentially expressed genes (Myt1l, Zmat4, Csrnp3, Dach2, Znf385b, Dlx6, Lhx8, Dlx1, Sp9), as shown in Figure 5B1, B2, and in Supplemental Table S2.

### Validation study with ISH histochemistry

**Figure 6:**
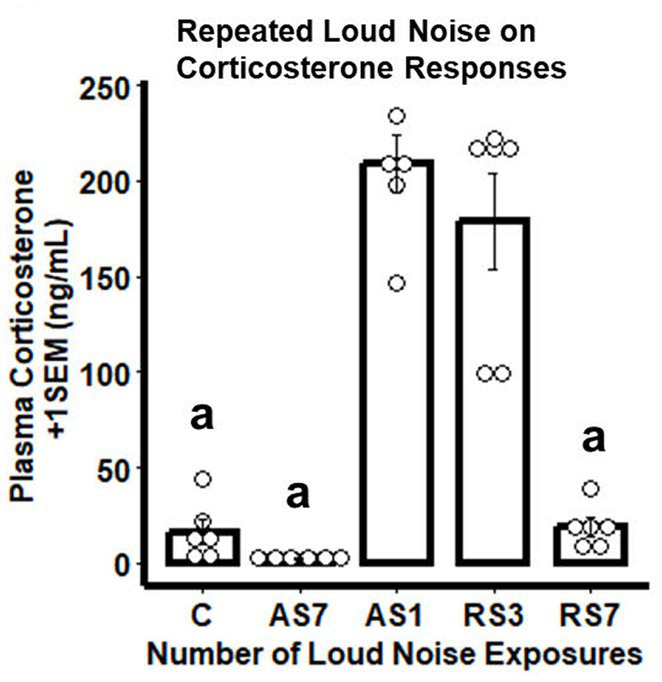
Combined bar/dotplots illustrating individual plasma corticosterone levels (ng/ml – white dots), group means (bars) and standard errors of the mean (SEM) achieved in the validation study in the control (C) and loud noise exposed rats [1(AS), 3(RS3) and 7(RS7) sessions of 30 min loud noise (100 dBA SPL; *n*=6/group)]. ^a^Denotes significant differences with the AS1 and RS3 groups (*post-hoc* comparisons with Bonferroni correction, *p* ≤ 0.05).

Similar to the CORT results of the RNA-seq study, rats experiencing the largest number of loud noise exposures (RS7) displayed the largest reduction in CORT levels on the last stress day, indicating significant habituation to the repeated loud noise exposures (Figure 6). The control (C), singly loud noise exposed 7 days prior to blood sampling (AS7) and the group exposed to 7 loud noises (RS7) all had low CORT levels, while a single loud noise exposure on the sampling day (AS1) was associated with the largest CORT response. Seven loud noise exposures reliably reduced CORT levels at the end of the 30 min noise exposure compared to the 1 (AS1) and 3 (RS3) loud noise-exposed groups. These observations were supported by a one-way ANOVA, indicating a significant effect of differential loud noise exposures (*F*_4,24_ = 46.03, *p* < 0.05). *Post-hoc* mean comparisons (Bonferroni correction) indicated that while the C, AS7 and RS7 groups did not differ from each other (all *p*’s > 0.05), they all significantly differed from the AS1 and RS3 groups (all *p*’s < 0.05), which did not differ from each other (*p* > 0.05).

**Figure 7:**
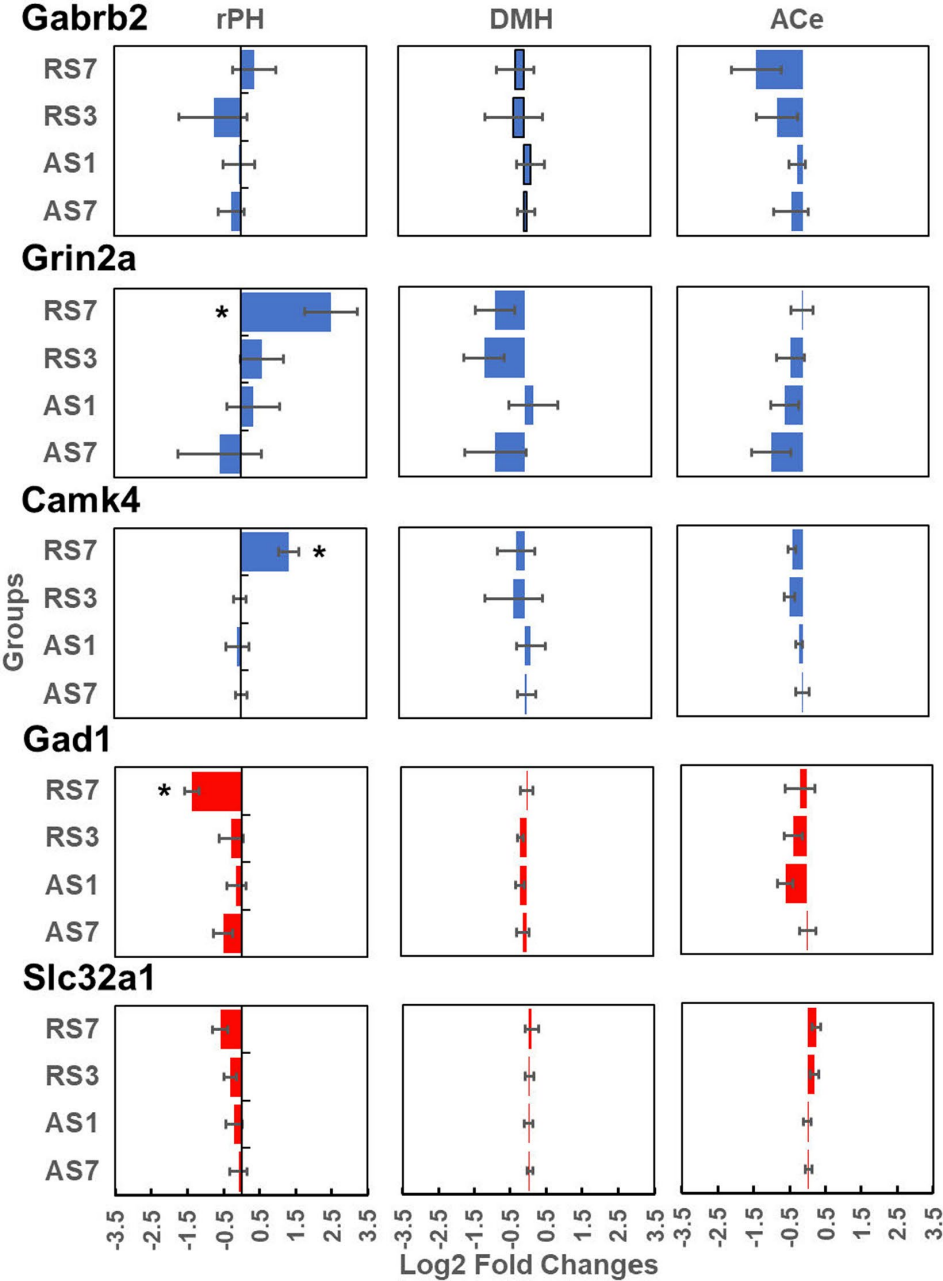
Log2 fold change values (±1 SEM) from radiometric *in situ* hybridization histochemical detection of Gabrb2 (top line panels), Grin2a (second line panels), Camk4 (third line panels), Gad1 (fourth line panel) and Slc32a (last line panels). These results are presented for the rostral posterior hypothalamic region (rPH; left hand panels), the dorsomedial hypothalamic nucleus (DMH; middle panels) and the central nucleus of the amygdala (ACe; right hand panels). Each panel includes the log2 fold changes of the AS7, AS1, RS3 and RS7 groups (*n* = 6/group) compared to their respective control groups for each brain region and transcript. *Denotes significant contrasts between the indicated groups and their respective control group (*p* ≤ 0.01).

**Figure 8:**
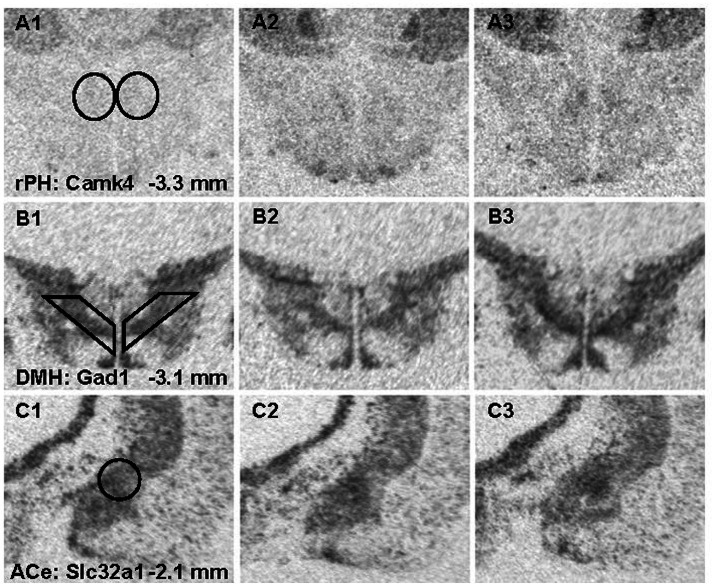
Representative photomicrographs from x-ray films exposed to brain sections hybridized with radioisotopic cRNA probes against Camk4 (A1-3), Gad1 (B1-3) and Slc32a1 (C1-3) mRNA across 3 different brain regions. Templates used for semi-quantitative analysis are shown in panel A1 for the rostral posterior hypothalamic nucleus (rPH) bilaterally; B1 for the dorsomedial hypothalamic nucleus (DMH) bilaterially; and C1 for the central nucleus of the amygdala (ACe) unilaterally. The left panels show representative sections from the no noise control (C) group (A-C1); the middle panels are from the group exposed to a single loud noise (AS1) 24-h prior to euthanasia (A-C2); and the right panels display sections from the group experiencing 7 loud noise (RS7) sessions (A-C3).

Radiometric ISH histochemistry was performed against three transcripts of significantly up-regulated (Gabrb2, Grin2a and Camk4) and two down-regulated (Gad1 and Slc32a) genes detected in the C versus RS7 comparison to begin to validate the RNA-seq results. Expression was examined in the hypothalamic rPH, DMH and forebrain ACe to assess specificity of differential gene expression. As shown in Figure 7, all of the transcripts assessed showed the expected changes in the rPH region, with up-regulation of the Gabrb2, Grin2a and Camk4 (blue) and down-regulation of Gad1 and Slc32a. Statistically, only the rPH Camk4 (contrast *t* value = 4.06, *p* < 0.01), Grin2a (contrast *t* value = 3.12, *p* < 0.01), and Gad1 (contrast *t* value = −2.53, *p* = 0.01) transcripts from the RS7 group were significantly different from their respective control group integrated density values. Polynomial contrasts were additionally performed in the groups receiving 1 (AS1), 3 (RS3) or 7 (RS7) loud noise exposures 24 h prior to euthanasia, reaching significance for Grin2a, Camk4 and Gad1 transcripts, (all *p*’s < 0.05), but not Gabrb2 or Slc32a (all *p*’s > 0.05). These results are similar to the findings of the RNA-seq study (Figure 3), suggesting that increasing homotypic stress exposures leads to larger differential gene expression. Pearson’s correlations revealed a similar pattern of results, indicating significant negative correlations between CORT and rPH Grin2a (*r*: −0.61, *t*_16_ = 3.04, *p* < 0.05) and rPH Camk4 (*r*: −0.62, *t*_16_ = 3.14, *p* < 0.05) mRNAs, and a positive correlation for rPH Gad1 (*r*: 0.52, *t*_16_ = 2.45, *p* < 0.05) mRNA; none of the other correlations for Gabrb2, Slc32a1 or other brain regions approached significance. The transcript regulation observed was therefore relatively specific to the rPH, as none of the contrasts or correlations in other groups or brain regions were statistically significant, as indicated in Figure 7. Examples of autoradiograms and regions quantified in the validation study are shown in Figure 8.

## Discussion

These studies are among the first to report significant gene regulation induced by exposure to repeated homotypic stress which typically leads to stress response habituation in the neuroendocrine, autonomic and behavioral domains (Watts, 1975; Zbrozyna, 1976; Abbott et al., 1984; Abercrombie and Jacobs, 1987; McCarty et al., 1992; Dobrakovova et al., 1993; Chen and Herbert, 1995; Yu and Blessing, 1997; Marti and Armario, 1998; Campeau et al., 2002; Carter et al., 2004; Grissom et al., 2008; Masini et al., 2008, 2011; Sasse et al., 2013). It should be noted that rats were deliberately euthanized 24 h after their last control or stress exposure at a time when relatively steady-state transcriptional responses should be achieved and maintained; this was suggested by the lack of immediate-early genes in our lists of differentially expressed genes at any time. However, the duration of the observed gene expression changes should ultimately be tested after different post-stress intervals given the reported gene profile changes at different intervals following chronic stress (Gray et al., 2014). Another important feature of the study designs employed differential stress exposures known to be associated with different phases of the habituation process; whereas one loud noise exposure was not expected to induce significant response habituation, 3 exposures were expected to induce a moderate reduction, while 7 exposures were expected to induce significant and sizable reduction in CORT release (Masini et al., 2008), which was observed in both studies. Interestingly, the steady-state transcriptional profiles of the differentially stressed groups tracked these CORT responses, in that no significant transcript regulation was observed after comparing the control group to the one loud noise group, a moderate gene number (36) after 3 exposures, and a large number (405) of differentially expressed genes after 7 exposures in the group displaying the largest CORT reduction in response to the same loud noise stimulus. These results were confirmed in large extents upon comparisons amongst the stressed groups, where comparisons with the one and three stress groups gave rise to few differentially expressed genes, but many differentially expressed genes between the 1 and 7 loud noise groups, without differences between the 3 and 7 stressor groups. These results especially suggest that the transcript regulation induced by 3 loud noises is intermediate, making it difficult to obtain significant gene regulation with either the control, 1 or 7 loud noise exposed groups. Furthermore, the lack of differentially expressed genes between the control and 1-stress groups led to the expectation that these 2 groups would comparably compare when contrasted with the 7 loud noise exposed group. This was indeed the case, as the reported numbers of differentially expressed genes was relatively large between the C, AS and RS7 groups (and similar to other studies (Surget et al., 2009)), but still small compared to the differential expression reported in other studies of chronic stress in forebrain regions (Gray et al., 2014; Bagot et al., 2016). This could be explained by differences in statistical thresholding strategies, the stress-to-tissue collection interval (1 h vs. 24-h or longer), the differences in physical stressor (forced swim/restraint vs. loud noise), the species (mice vs. rats) or the brain regions (hippocampus/prefrontal cortex/nucleus accumbens vs. hypothalamus) investigated. The magnitude of the fold changes also tended to be smaller in the current study compared to prior studies (Reyes et al., 2003; Gray et al., 2014; Bagot et al., 2016). However, it is difficult to anticipate the relationship between mRNA fold changes and the functional neural modifications induced in these experiments without concurrently examining readouts at the protein level, for example by incorporating immunohistochemical or immunoblotting approaches.

Slightly over 50% of the genes reported after the control versus 3 stress exposures (17/33) group comparison were also differentially expressed after 7 stress exposures, but larger differences from the control group were typically obtained with 7 stress exposures. A relatively similar pattern of results was obtained between the 1, 3 and 7 stress exposed groups, respectively. It is interesting to note that from learning theory, the largest learning changes are generally believed to take place upon initial stimulus presentation and get incrementally smaller with repeated learning trials, whether the learning is associative (Rescorla and Wagner, 1972) or non-associative (Groves and Thompson, 1970). This apparent discrepancy most likely reflects that the 24-h interval after the first loud noise missed the initial transcriptional response that is rather large 1-h after a stress episode and recruits multiple immediate-early genes (Reyes et al., 2003; Gray et al., 2014). Thus, while the initial transcriptional responses to stress are large in the forebrain and hypothalamus 1-h after stress (Reyes et al., 2003; Gray et al., 2014), these responses are already reduced 3-h after stress (Reyes et al., 2003), and our RNA-seq study did not identify significant changes 24-h after a single stressor when compared to the control group. It is thus conceivable that transcriptional responses get incrementally smaller to repeated homotypic stress, while the steady-state changes may get increasingly larger over multiple exposures. Future studies could examine this dichotomy by sampling transcriptional responses sooner (1-h) after differential stressor exposures with the use of appropriate controls to factor in the steady-state changes. Such a design, with the help of nascent-transcript profiling (such as Global Run-On sequencing), might shed light on the distinction between transient and sustained/steady-state transcriptional responses associated with repeated homotypic stress exposures.

These experiments are also among the first to explore one of the more proximal regions that may provide direct control over multiple effector response systems activated by stress experiences (Stotz-Potter et al., 1996; Morin et al., 2001; Myers et al., 2016; Nyhuis et al., 2016) in the context of gene regulation. Focus on the rostral posterior hypothalamic region was further dictated by prior findings demonstrating that normal rPH neural activity interference significantly hindered the development of neuroendocrine habituation to repeated loud noise or restraint stress (Nyhuis et al., 2016). Whether intrinsic regulation of gene expression in the rPH or connected brain regions underlie the development of repeated homotypic stress response habituation was not directly tested in this study. The present results provide evidence that intrinsic posterior hypothalamic gene regulation is associated with, and thus could contribute to, the development of homotypic stress response habituation. Indeed, gene ontology analyses were consistent with significant regulation of transcripts associated with neuron differentiation, neural membrane potential, pre- and post-synaptic elements, chemical synaptic transmission, vesicles, axon guidance and projection, glutamatergic and GABAergic synapses. Many of these terms have also been reported following chronic unpredictable stress in the forebrain of prior studies (Surget et al., 2009; Gray et al., 2014; Bagot et al., 2016). No significant enrichment of immune-related terms was obtained in this study, whereas other studies employing chronic unpredictable stress reported significant transcriptional regulation of immune-related gene activation (Gray et al., 2014; Malki et al., 2015). This suggests that habituated regimens of repeated homotypic stressors may recruit transcriptional responses distinct from chronic unpredictable stress paradigms. To summarize, gene enrichment of the biological and cellular functions in the current study firmly point to a significant reorganization of the rPH region that could in turn mediate the phenotypic modification associated with repeated homotypic stress habituation. Proof of the necessity or sufficiency of rPH transcriptional plasticity in stress habituation awaits direct manipulation of identified candidate target genes, for example using knockdown or genome editing approaches.

In order to help zero in on potential signals that might drive differential regulation of genes identified in the comparisons between the control, 1 and 7 loud noise-exposed groups respectively, transcription factor enrichment analyses were performed and revealed several transcription factors that could account for some of the rPH regulation observed. At the top of the list were transcription factors (Csrnp3, Myt1l, Znf365 and Zmat4) associated with roughly one third (48–97) of the upregulated transcripts. There was significant overlap between the gene lists associated with these transcription factors, but interestingly, Csrnp3, Myt1l and Zmat4 were also up-regulated genes reported from the differential expression analysis of either the C or AS versus RS7 comparison lists. Components of these gene lists included members of the GABA_A_ receptor subunit family, glutamatergic ionotropic receptor subunit family, potassium and calcium channels regulating membrane potentials, synaptic machinery, and intracellular signaling systems. Another promising finding was Myt1l, which encodes a myelin transcription factor. Other groups have reported regulation of oligodendrocytes/myelination by chronic stress in forebrain regions (Surget et al., 2009; Liu et al., 2012, 2018). In contrast to the down-regulation of oligodendrocytic/myelin-associated genes with chronic unpredictable stress in the forebrain, however, repeated homotypic stress was associated with up-regulation of this gene family in the rPH. Given the mounting evidence of the role of oligodendrocytes in adult learning and plasticity (Hughes and Appel, 2016; Hughes E. G. et al., 2018; Bacmeister et al., 2022), understanding mechanisms and targets of induction of this pathway may be highly relevant in the context of habituation to repeated homotypic stress. From the down-regulated gene lists, Dlx6, Dlx1, Lhx8 and Sp9 were at the top of the transcription factors associated with moderate numbers of genes (9–22) from both the C or AS versus RS7 comparison lists and included GABA neurotransmitter enzymes Gad1 and 2, and the vesicular GABA transporter Slc32a1 (Vgat). Dlx5/6 are exclusively located in GABAergic neurons and subject to regulation by Foxp2 (Levi et al., 2021), which was one of the up-regulated genes reported in the C versus RS3, C versus RS7, and AS versus RS7 group comparison lists. Several of these transcription factors could thus feasibly contribute to a significant proportion of the observed transcriptional regulation in the rPH and provide promising targets for future studies of transcriptional mediators of homotypic stress response habituation.

To validate some of the differentially expressed gene results, an entirely independent study was performed with *in situ* hybridization histochemistry against some of the transcripts that could significantly contribute to functional modifications in rPH, as revealed in the RNA-seq study. This validation study added a control group to determine if the effects of a single loud noise exposure might induce significant gene regulation when observed at a later interval (7 days instead of 1 day) and might account for the apparent larger number of differentially expressed genes in the group exposed to 7 loud noises. In addition to sampling the rPH region, transcripts in the closely located dorsomedial hypothalamic nucleus and the forebrain central nucleus of the amygdala were quantified to assess the spatial specificity of putative changes observed in the rPH. All the transcripts measured (Gabrb2, Grin2a, Camk4, Gad1 and Slc32a1) displayed changes concordant with the RNA-seq results, with 3 of these measurements (Camk4, Grin2a and Gad1) reaching statistical significance in the group exposed to 7 loud noises. Although not significant from the *in situ* hybridization results, the Gabrb2 transcript displayed a tendency to be up-regulated in the rPH region, in agreement with the RNA-seq results, but interestingly, this is opposite to the down-regulation previously reported in the paraventricular hypothalamic nucleus in response to a non-habituating chronic stress paradigm (Cullinan and Wolfe, 2000). None of the contrasts reached significance in the other brain regions, suggesting that the differential gene expression induced by a repeated homotypic stress regimen in the rPH was specific. This, however, does not rule out important changes in the DMH or ACe, but rather implies that regulation in these regions would likely recruit different patterns of gene regulation. In further agreement with the RNA-seq results, most of the measured transcripts, except for Gabrb2, were incrementally modified with increasing loud noise exposures in the rPH when compared to the no noise control group. Finally, there was very little support for the possibility that a single loud noise experienced 7 days prior to euthanasia induced the same level of transcriptional regulation induced in the group exposed to 7 loud noises.

In summary, as informed by prior studies investigating forebrain transcriptional regulation in the context of repeated stress (Surget et al., 2009; Gray et al., 2014; Ding et al., 2015; Malki et al., 2015; Bagot et al., 2016, 2017), we report a significant transcriptional response in the rPH region. However, while most studies have focused on chronic variable or unpredictable non-habituating stress due to their more apparent associations with psychiatric or psychologic disorders (Radley et al., 2015), we demonstrate that a repeated homotypic stress regimen is effective in inducing a strong transcriptional response that correlates with the development of neuroendocrine habituation. These results support prior work suggesting that the posterior hypothalamic region significantly contributes to the development of repeated homotypic stress habituation (Nyhuis et al., 2016). Importantly, the transcriptional patterns obtained with repeated homotypic stress exposures differ in important ways from those reported by multiple studies employing chronic non-habituating stress procedures (Surget et al., 2009; Gray et al., 2014; Ding et al., 2015; Malki et al., 2015; Bagot et al., 2016, 2017); there are significant differences in the activation of immune-related transcripts, and opposite regulation of oligodendrocytic/myelin gene expression. Whereas there are apparent commonalities in the regulation of multiple cellular functions, including the glutamate and GABAergic neurotransmitter systems, a more detailed look at these systems will be required to assess their functional similarities or differences within specific circuits. It will be important to examine immediate and steady-state transcriptional responses in the context of habituating protocols of repeated homotypic stress versus non-habituating chronic heterotypic stressors to help tease apart their differential effects in stress-responsive brain circuits and on behavior. Such studies could provide important clues about the crucial signals that distinguish these transcriptional events. An additional limitation of the current study was the lack of assessment of transcriptional regulation across sex; future studies should assess potential sex effects in these transcriptional responses, given the role of gender in the incidence of mood disorders. These results also provide credibility to the hypothesis that the hypothalamus is an integral component of circuits that are critical to socioemotional behaviors (Caria, 2023), and are not simply passive recipients of forebrain-controlled processing. More attention might be required to the possibility that direct regulation of hypothalamic circuits including the rPH contribute significantly to adaptive versus maladaptive regulation associated with affective disorders (Campeau et al., 2011; Radley et al., 2015).

## Data availability statement

The datasets presented in this study can be found in online repositories. The names of the repository/repositories and accession number(s) can be found at: https://www.ncbi.nlm.nih.gov/geo/, GSE229159.

## Ethics statement

The animal study was reviewed and approved by Institutional Animal Care and Use Committee of the University of Colorado Boulder, Protocol #2573.

## Author contributions

SC and RD provided the conception, designs, and intellectual content of these studies. SS, AG, CM, JS, and SC performed the data acquisition and analyses. SC, CM, JS, SS, and RD performed the drafting and revision of the manuscript. All authors contributed to the article and approved the submitted version.

## Funding

This work was supported by a University of Colorado Boulder Research and Innovation seed grant to SC and RD.

## Conflict of interest

The authors declare that the research was conducted in the absence of any commercial or financial relationships that could be construed as a potential conflict of interest.

## Publisher’s note

All claims expressed in this article are solely those of the authors and do not necessarily represent those of their affiliated organizations, or those of the publisher, the editors and the reviewers. Any product that may be evaluated in this article, or claim that may be made by its manufacturer, is not guaranteed or endorsed by the publisher.

## Footnotes

^1^ https://ftp.ensembl.org/pub/release-100/gtf/rattus_norvegicus/Rattus_norvegicus.Rnor_6.0.100.gtf.gz

^2^ https://david.ncifcrf.gov/home.jsp

^3^ https://maayanlab.cloud/chea3/
