# Supplementary material for: Determination of steady-state transcriptome modifications associated with repeated homotypic stress in the rat rostral posterior hypothalamic region
Source: Front Neurosci. 2023 Jun 9;17:1173699. doi: 10.3389/fnins.2023.1173699 (PMC10288150; doi:10.3389/fnins.2023.1173699)
Supplement: Supplementary file 1 [file Data_Sheet_1.pdf]

Supplementary Material

Determination of steady-state transcriptome modifications associated with repeated homotypic stress in the rat rostral posterior hypothalamic region.

Serge Campeau*, Connor McNulty, Jacob T. Stanley, Anthony N. Gerber, Sarah K. Sasse, and Robin D. Dowell

*** Correspondence:** serge.campeau@colorado.edu

Supplemental Table S1. Lists of genes reported significant with DESeq2 differential expression analysis between the Control vs. 3 Loud noise exposures, Control vs. 7 Loud noise exposures, 1 vs. 3 Loud noise exposures, and 1 vs. 7 Loud noise exposures, respectively. The gene lists are organized from highest negative to highest positive log2 fold changes. For each gene, their symbol, Ensembl gene number, baseMean, log2 fold change (lfc), log2 fold change standard error (lfcSE), and the Benjamini-Hochberg P adjusted (*Padj*) value obtained following the Wald test statistics in DESeq2 are provided.

Control vs. 3 Loud noise exposures

Gene symbol	Ensembl gene	baseMean	log2FoldChange	lfcSE	*Padj*
Gad2	ENSRNOG00000018200	4964.640114	-0.53576916	0.131189852	0.037906636
Gucy1a1	ENSRNOG00000012302	440.6497415	-0.414872585	0.108918439	0.037906636
Ino80e	ENSRNOG00000019960	428.0281556	-0.367652062	0.095491365	0.037906636
Nr2f2	ENSRNOG00000010308	780.177892	-0.320889144	0.084689853	0.037906636
Eif3c	ENSRNOG00000018761	1999.274207	-0.310520742	0.084637939	0.046002789
Tfg	ENSRNOG00000001633	799.9527957	-0.297442278	0.08136726	0.046002789
Aqp4	ENSRNOG00000016043	11883.22853	-0.297057256	0.077587599	0.037906636
Ccar1	ENSRNOG00000000397	856.4937564	-0.277281744	0.071400086	0.037906636
Col4a1	ENSRNOG00000016281	660.0820336	-0.275537286	0.071319018	0.037906636
Acin1	ENSRNOG00000013533	1905.233492	-0.242137661	0.064659081	0.040124118
Phyhipl	ENSRNOG00000000274	2336.800578	-0.229666591	0.063748674	0.048614996
Utrn	ENSRNOG00000011058	1465.77435	-0.21704794	0.057583278	0.037906636
Marcks	ENSRNOG00000000579	2748.035305	-0.212773282	0.058948726	0.048614996
Rnf187	ENSRNOG00000049219	3566.033107	0.167544959	0.045504182	0.045932595
Fkbp8	ENSRNOG00000058359	2383.599097	0.18130828	0.049562378	0.046002789
Rnf187	ENSRNOG00000007604	1229.261606	0.189194354	0.052454275	0.048614996
Psmd13	ENSRNOG00000014109	769.69618	0.198645666	0.053314202	0.041589132
Ip6k1	ENSRNOG00000019932	930.8188961	0.246572944	0.065136478	0.037906636
Stmn1	ENSRNOG00000016810	3714.454517	0.257584746	0.067819569	0.037906636
Cope	ENSRNOG00000020178	466.4592121	0.272464235	0.072281814	0.037906636
Slc36a4	ENSRNOG00000011455	779.7040538	0.284728632	0.075064587	0.037906636
Pgk1	ENSRNOG00000058249	1555.454491	0.296650546	0.075668873	0.037906636
Cox7a2	ENSRNOG00000027791	864.4797137	0.313472038	0.086143966	0.047542049

Gene symbol	Ensembl gene	baseMean	log2FoldChange	lfcSE	*Padj*
Wbp4	ENSRNOG00000011678	469.9186027	0.337399598	0.083355756	0.037906636
Atxn7l3	ENSRNOG00000020930	884.9030417	0.341195589	0.091968268	0.042669273
Abhd8	ENSRNOG00000000054	1938.21155	0.352288631	0.085235143	0.037906636
Cndp2	ENSRNOG00000015591	1227.295408	0.355397337	0.089825449	0.037906636
Foxp2	ENSRNOG00000054508	803.8614382	0.415910569	0.109657919	0.037906636
Tp53i11	ENSRNOG00000008738	818.4093613	0.46029269	0.104110622	0.030132217
Necab3	ENSRNOG00000016708	876.9268937	0.487048261	0.123663958	0.037906636
Lypd6	ENSRNOG00000038980	486.0666468	0.522273923	0.129968767	0.037906636
Prkcq	ENSRNOG00000019057	532.9662267	0.523107486	0.120640837	0.030132217
Wnt4	ENSRNOG00000013166	461.3861389	0.595127171	0.138055217	0.030132217
NA	ENSRNOG00000057759	692.4420314	-0.248037807	0.059928679	0.037906636
NA	ENSRNOG00000011955	630.6543946	0.258185337	0.066783816	0.037906636
LOC684970	ENSRNOG00000021986	681.2496194	-0.190752938	0.052892145	0.048614996

Control vs. 7 Loud noise exposures

Gene symbol	Ensembl gene	baseMean	log2FoldChange	lfcSE	*Padj*
Prlhr	ENSRNOG00000009922	24.58690572	-1.946573538	0.57715552	0.035850089
Isl1	ENSRNOG00000012556	99.95575544	-1.286373727	0.389348304	0.040292516
Dlx5	ENSRNOG00000010905	33.53698637	-1.243790329	0.323186838	0.014988691
Gal	ENSRNOG00000015156	122.5956986	-1.212544531	0.372558528	0.045319586
Calcr	ENSRNOG00000010053	375.2178082	-1.207518561	0.337461762	0.023780271
Agtr1a	ENSRNOG00000018346	18.93031004	-1.179939674	0.337405333	0.028038623
Ankdd1a	ENSRNOG00000015554	18.22485272	-1.155213236	0.280679858	0.009095412
Scn5a	ENSRNOG00000015049	130.910339	-1.078611281	0.199741425	0.00016489
Crhr2	ENSRNOG00000011145	34.70884024	-1.075191771	0.277542025	0.014721542
Krt77	ENSRNOG00000036865	23.00426612	-1.051721143	0.277685361	0.016000214
Sim1	ENSRNOG00000037600	113.8388682	-0.969912444	0.214393977	0.003003934
Dlx6	ENSRNOG00000010822	27.74606679	-0.937297648	0.232201251	0.011343237
Gck	ENSRNOG00000061527	40.69867367	-0.92912812	0.260497677	0.024218402
Kcng1	ENSRNOG00000054314	30.42962858	-0.918897199	0.235992806	0.014594676
Esyt3	ENSRNOG00000022704	63.20203089	-0.884873617	0.263102732	0.036386727
Penk	ENSRNOG00000008943	595.6618097	-0.868268837	0.22506958	0.014988691
Arhgap36	ENSRNOG00000007552	367.5291032	-0.862471719	0.218869454	0.013876716
Cckbr	ENSRNOG00000017679	36.52751081	-0.858636855	0.251748869	0.033572986
Slc12a3	ENSRNOG00000057072	30.86652967	-0.847092018	0.219552612	0.014988691
Myh7	ENSRNOG00000016983	28.30903191	-0.826996256	0.251252416	0.041367357
Mr1	ENSRNOG00000003522	46.11779071	-0.788088609	0.210651849	0.017296157
Gpc3	ENSRNOG00000060179	133.191582	-0.74826943	0.221276385	0.035432844
Fam227a	ENSRNOG00000021795	169.9103355	-0.741040957	0.211477828	0.027898755
Pla2g4b	ENSRNOG00000007447	70.75185992	-0.737841576	0.158723722	0.002067792
Lhx5	ENSRNOG00000001392	51.6589484	-0.726719645	0.210369706	0.031247429
Fam20a	ENSRNOG00000003969	61.19431207	-0.717339754	0.18266396	0.013876716
Hspa1a	ENSRNOG00000050647	42.96571151	-0.69765435	0.184642116	0.016162918
Lrrc36	ENSRNOG00000016854	46.68029013	-0.68768588	0.212506005	0.046983522
Xkr6	ENSRNOG00000011634	68.19405865	-0.686083182	0.196199306	0.028038623
Ak9	ENSRNOG00000037688	107.2428623	-0.662488819	0.16592409	0.01212403

Gene symbol	Ensembl gene	baseMean	log2FoldChange	lfcSE	*Padj*
Greb1	ENSRNOG00000024651	83.10707709	-0.651135701	0.135508687	0.00120869
Cdh23	ENSRNOG00000033087	168.7558001	-0.637162971	0.165041248	0.014988691
Rsph10b	ENSRNOG00000001036	153.3416193	-0.630898129	0.195224644	0.047592904
Gad1	ENSRNOG00000000007	2773.191849	-0.627005556	0.124140854	0.000594038
Nrp2	ENSRNOG00000031232	302.0202723	-0.624007939	0.162340147	0.014988691
Rxrg	ENSRNOG00000004537	108.8990897	-0.613131556	0.162821441	0.016643171
Gad2	ENSRNOG00000018200	4964.640114	-0.600668824	0.131171818	0.002389365
Cd24	ENSRNOG00000000321	215.026978	-0.59553854	0.176670308	0.035887698
Lgals3bp	ENSRNOG00000003217	143.0836203	-0.592352806	0.18153717	0.044484822
Tchh	ENSRNOG00000056746	51.96013666	-0.59078256	0.172986042	0.033483408
Lama3	ENSRNOG00000011300	93.38245871	-0.578894888	0.163906717	0.025963999
Syt6	ENSRNOG00000019163	330.8232362	-0.570167422	0.155785512	0.019209501
Rtbdn	ENSRNOG00000043215	64.095133	-0.564499578	0.171485752	0.041367357
Shisa7	ENSRNOG00000016877	650.6173505	-0.553566319	0.138449629	0.011990641
Rerg	ENSRNOG00000027592	151.204491	-0.54804867	0.154485871	0.025322089
Il18bp	ENSRNOG00000020150	59.0977789	-0.540188474	0.149528172	0.021535929
L3mbtl1	ENSRNOG00000007044	188.3066513	-0.535844161	0.131481601	0.010179155
Nnat	ENSRNOG00000024923	5211.522468	-0.530457338	0.15805954	0.036686935
Gucy1a1	ENSRNOG00000012302	440.6497415	-0.525808847	0.108867808	0.0011275
Magel2	ENSRNOG00000010158	324.0697689	-0.505406624	0.144903618	0.028688275
Sytl5	ENSRNOG00000003585	73.74314522	-0.499919004	0.152935901	0.043816437
Crocc	ENSRNOG00000008334	226.0387012	-0.499877149	0.127254566	0.013876716
Gpr165	ENSRNOG00000012995	1223.537458	-0.477851345	0.122636372	0.014594676
Cpne5	ENSRNOG00000000522	451.0357534	-0.474788098	0.146190491	0.046021784
Sbk1	ENSRNOG00000057696	119.9932536	-0.469184705	0.124548443	0.016643171
Ngef	ENSRNOG00000016653	401.5762393	-0.468582395	0.129226979	0.020745284
Fchsd1	ENSRNOG00000039415	134.8557917	-0.465896871	0.127073729	0.0189295
Scn9a	ENSRNOG00000006639	521.0385156	-0.461097218	0.136437593	0.035464429
Lmo3	ENSRNOG00000047450	414.7933161	-0.456720358	0.121301313	0.016643171
Ucp2	ENSRNOG00000017854	292.9891448	-0.446670572	0.121540847	0.018486492
P3h3	ENSRNOG00000016071	464.1275844	-0.424029797	0.110263741	0.014988691
Dynlt1	ENSRNOG00000018207	113.8222875	-0.423202939	0.131685257	0.049100398
Slc32a1	ENSRNOG00000015393	1070.89168	-0.419164739	0.126846057	0.040292516
Unc5a	ENSRNOG00000059840	263.3503278	-0.414587766	0.115312851	0.022612075
Npl	ENSRNOG00000002775	159.7092204	-0.410328093	0.109468861	0.017052265
Zfpm2	ENSRNOG00000004109	143.2607508	-0.408958735	0.122754107	0.038602826
Cadps2	ENSRNOG00000007636	542.0761601	-0.408671216	0.125342676	0.044765432
Pnma3	ENSRNOG00000052022	747.0376837	-0.405500829	0.114033874	0.024964194
Vstm2a	ENSRNOG00000005180	352.6361701	-0.399843681	0.118721792	0.036163654
Spry3	ENSRNOG00000060153	134.2694838	-0.396140299	0.117476466	0.035850089
Cd38	ENSRNOG00000003069	372.9048967	-0.395260118	0.106713967	0.017809762
Hap1	ENSRNOG00000014819	4275.232858	-0.395069083	0.122952667	0.049100398
Abhd14b	ENSRNOG00000012073	278.6129597	-0.38601005	0.117950435	0.043476298
Efnb2	ENSRNOG00000014648	237.0078297	-0.372782359	0.112351137	0.039594758
Myo16	ENSRNOG00000016483	307.9017809	-0.360608067	0.095128849	0.016000214
Zfta	ENSRNOG00000032042	259.2682018	-0.354735123	0.102737183	0.031319339
Fam104b	ENSRNOG00000049908	187.9195855	-0.353227828	0.090659514	0.014594676

Gene symbol	Ensembl gene	baseMean	log2FoldChange	lfcSE	*Padj*
Npr2	ENSRNOG00000015991	568.1890562	-0.349477469	0.095422578	0.019123422
Caprin2	ENSRNOG00000047319	212.197506	-0.345550555	0.103508242	0.038265163
Dvl2	ENSRNOG00000017915	154.9577928	-0.343517586	0.101262192	0.034761654
Plk2	ENSRNOG00000011951	324.2954796	-0.336174382	0.085510753	0.013876716
Nynrin	ENSRNOG00000048431	278.5380353	-0.336121575	0.100452223	0.037679483
Epb41l4a	ENSRNOG00000026050	235.0246898	-0.334245765	0.088032724	0.016000214
Ntrk3	ENSRNOG00000018674	406.8922875	-0.326907278	0.086168739	0.016000214
Zfp384	ENSRNOG00000017066	592.9750031	-0.311177369	0.08690938	0.023686745
Phactr1	ENSRNOG00000014264	378.5728792	-0.307210947	0.079145339	0.014594676
Zfp958	ENSRNOG00000030410	248.85786	-0.303614576	0.084020814	0.021535929
Zfp383	ENSRNOG00000046305	172.8435257	-0.298068564	0.089978738	0.03994529
Cnr1	ENSRNOG00000008223	1156.93244	-0.294139312	0.085875045	0.033325221
Nsmaf	ENSRNOG00000010234	288.2037765	-0.290970391	0.074885684	0.014594676
Msn	ENSRNOG00000030118	623.5590016	-0.290512438	0.068606372	0.007139075
Ctbp2	ENSRNOG00000017326	332.7078269	-0.289894391	0.077661434	0.017296157
Rasgef1a	ENSRNOG00000031671	427.0592169	-0.279334449	0.075735232	0.018131821
Cotl1	ENSRNOG00000016257	493.085032	-0.259394992	0.069005046	0.016660598
Sgsm1	ENSRNOG00000000708	1772.345909	-0.259017891	0.076642453	0.035464429
Dusp18	ENSRNOG00000024945	388.3646989	-0.254241107	0.077708681	0.043487263
Bicral	ENSRNOG00000016229	571.8908693	-0.246745171	0.068862228	0.023553537
Optn	ENSRNOG00000017941	1444.000238	-0.243178043	0.068524958	0.025322089
Snhg11	ENSRNOG00000036802	4995.792109	-0.23405119	0.069690761	0.036603255
Trnau1ap	ENSRNOG00000055344	285.2444501	-0.232547535	0.068958167	0.035850089
Col4a1	ENSRNOG00000016281	660.0820336	-0.227312833	0.070798409	0.049330544
Csk	ENSRNOG00000019374	429.7270018	-0.226356922	0.065045543	0.029217774
Slx4	ENSRNOG00000024445	501.326806	-0.215037036	0.059265261	0.020727709
Cstf2	ENSRNOG00000048025	1299.437077	-0.212291718	0.063624365	0.038272127
MGC109340	ENSRNOG00000046858	1190.582637	-0.205605146	0.054189474	0.016000214
Stk38	ENSRNOG00000000519	525.3055104	-0.198845528	0.058857347	0.035491801
Iqsec3	ENSRNOG00000014083	2238.608215	-0.193131935	0.053209298	0.020727709
Marcks	ENSRNOG00000000579	2748.035305	-0.18843089	0.05881092	0.04982643
Specc1l	ENSRNOG00000001303	867.7345529	-0.185676089	0.05005277	0.017714812
Utrn	ENSRNOG00000011058	1465.77435	-0.184319978	0.057317759	0.049100398
Ddx39b	ENSRNOG00000000841	2682.345211	-0.176963866	0.051684097	0.033325221
Tnrc6a	ENSRNOG00000024737	2361.303639	-0.166728654	0.048800942	0.033483408
Syt11	ENSRNOG00000020279	14835.3699	0.105420251	0.032725215	0.048698719
Pkm	ENSRNOG00000011329	2834.584464	0.127813372	0.03759499	0.034066221
Bltp2	ENSRNOG00000011887	3207.063588	0.130237424	0.03829579	0.034066221
App	ENSRNOG00000006997	22609.58077	0.14493343	0.040830936	0.025322089
Jph3	ENSRNOG00000018784	2780.525771	0.147889626	0.043991111	0.036386727
Srcin1	ENSRNOG00000011475	1596.364001	0.148563042	0.042356384	0.027760122
Eps15	ENSRNOG00000010299	2191.365716	0.155941653	0.044008272	0.025607055
Ypel3	ENSRNOG00000019721	1124.740919	0.159592962	0.049811688	0.04982643
Hectd1	ENSRNOG00000006905	2600.411911	0.160209068	0.041622989	0.014988691
Fam168b	ENSRNOG00000023467	2349.302549	0.162107433	0.043755041	0.017809762
Tspyl4	ENSRNOG00000000547	3733.667919	0.162174756	0.042420174	0.015763733

Gene symbol	Ensembl gene	baseMean	log2FoldChange	lfcSE	*Padj*
Herc2	ENSRNOG00000013718	4164.297108	0.164180706	0.046769647	0.027567001
Adam23	ENSRNOG00000012424	1351.981765	0.165811117	0.050889643	0.044868027
Rab11fip5	ENSRNOG00000056009	1265.721819	0.169668268	0.049612642	0.033448273
Usp32	ENSRNOG00000027711	3433.784894	0.169848599	0.048551324	0.028038623
Prkcz	ENSRNOG00000015480	1840.899066	0.173910134	0.051138081	0.034066221
Synj1	ENSRNOG00000002051	6901.43727	0.174170501	0.051250803	0.034118642
Pxk	ENSRNOG00000008024	837.8443859	0.176885645	0.053470615	0.040128774
Clasp1	ENSRNOG00000002376	2240.17196	0.177300318	0.045943634	0.014988691
Dync1i1	ENSRNOG00000009700	2119.922619	0.182363409	0.056840243	0.049434508
Wwc1	ENSRNOG00000008065	1080.59409	0.185683564	0.055808518	0.03877432
St6galnac6	ENSRNOG00000046984	1253.886588	0.185769602	0.050538352	0.018486492
Akap11	ENSRNOG00000009987	4012.864465	0.186968752	0.047503196	0.013876716
Lynx1	ENSRNOG00000006086	3807.445247	0.187624403	0.049293048	0.016000214
Trim37	ENSRNOG00000006248	2208.743527	0.189245281	0.057817673	0.043476298
Ciao1	ENSRNOG00000012638	475.0303479	0.19609049	0.057151943	0.03282482
Ralgapa1	ENSRNOG00000046256	1589.596998	0.197788233	0.055510117	0.024404135
Ipo5	ENSRNOG00000010989	1257.789836	0.198332419	0.056972749	0.029217774
Ppia	ENSRNOG00000027864	1282.177074	0.199619281	0.053579924	0.017417348
Herc1	ENSRNOG00000051671	3774.818523	0.203636014	0.059182962	0.032137181
Napb	ENSRNOG00000004753	1505.138774	0.204038911	0.057087489	0.023933826
Gpcpd1	ENSRNOG00000053201	1212.577074	0.204731925	0.063037496	0.046021784
Prkcb	ENSRNOG00000012061	1599.431495	0.205856678	0.059157299	0.029217774
Efr3b	ENSRNOG00000012950	1857.75774	0.206127136	0.060378774	0.033483408
Acaca	ENSRNOG00000034013	1513.619899	0.208371859	0.06450101	0.047657527
Cask	ENSRNOG00000060946	563.9160555	0.208545898	0.062838979	0.039594758
Usp15	ENSRNOG00000023202	727.7561593	0.213725037	0.06335226	0.035850089
Tgfb2	ENSRNOG00000002418	536.048234	0.215181821	0.06692964	0.049100398
Calm1	ENSRNOG00000004060	14043.36411	0.215423675	0.063352954	0.034066221
Fbxl16	ENSRNOG00000022248	1358.021647	0.216082811	0.062415564	0.030621666
Clstn1	ENSRNOG00000016398	11403.54336	0.21884218	0.068085352	0.049100398
Sptbn2	ENSRNOG00000058842	4045.212088	0.21890785	0.059295898	0.018069007
Pcdh10	ENSRNOG00000031974	1270.613946	0.218911067	0.066268092	0.040292516
Igsf8	ENSRNOG00000007604	1229.261606	0.219324466	0.052148698	0.007232022
Pcsk6	ENSRNOG00000011526	458.3423759	0.221551639	0.064737269	0.033404458
Snx16	ENSRNOG00000009953	484.9002514	0.222539082	0.065774665	0.035324756
Opa1	ENSRNOG00000001717	1657.561893	0.223911288	0.045070173	0.000627615
Gpr158	ENSRNOG00000024832	1456.354533	0.225676981	0.069598018	0.046623932
Oxr1	ENSRNOG00000056487	6024.225062	0.226339472	0.062167529	0.020383131
Ncdn	ENSRNOG00000011751	13030.21511	0.227769277	0.069019961	0.040548608
Ehd3	ENSRNOG00000007744	775.7697662	0.228912309	0.071303517	0.049330544
Cdh8	ENSRNOG00000056643	1174.371839	0.232771767	0.062360267	0.017296157
Sptbn1	ENSRNOG00000005434	19121.17448	0.233699757	0.067279342	0.029558496
Kcnq2	ENSRNOG00000011624	1801.61533	0.234180318	0.068493346	0.033448273
Rap1gap	ENSRNOG00000013825	3291.465813	0.23631052	0.069720298	0.034902007
Vsnl1	ENSRNOG00000005345	7693.908656	0.23640344	0.062265855	0.016000214
Hsph1	ENSRNOG00000000902	6562.373546	0.23727247	0.072091336	0.041367357
Miga1	ENSRNOG00000056332	702.8130921	0.240776224	0.054438076	0.004130897

Gene symbol	Ensembl gene	baseMean	log2FoldChange	lfcSE	*Padj*
Adgrl2	ENSRNOG00000032660	1161.569561	0.244668745	0.065199976	0.016875534
Bltp1	ENSRNOG00000038436	3835.515925	0.245070922	0.060541565	0.010958987
Septin5	ENSRNOG00000029912	3350.18487	0.249868787	0.071310878	0.027898755
Dnajc6	ENSRNOG00000052887	6307.038448	0.25137309	0.066345181	0.016000214
Rnaseh2a	ENSRNOG00000003504	366.5983932	0.254474627	0.079113347	0.049100398
Trps1	ENSRNOG00000024998	307.7646326	0.257596958	0.073230119	0.027051766
Coro2b	ENSRNOG00000015257	1158.633068	0.258178561	0.078633806	0.042319052
Mib1	ENSRNOG00000013281	848.8008623	0.258293154	0.061671647	0.007585769
Rhob	ENSRNOG00000021403	5676.400003	0.258850866	0.062865452	0.009095412
Tnks2	ENSRNOG00000059776	356.2359128	0.261014257	0.080590334	0.046785136
Purb	ENSRNOG00000056150	1125.913794	0.262329404	0.076966452	0.033572986
Usp31	ENSRNOG00000025793	1000.931292	0.262987233	0.078733171	0.038245429
Rictor	ENSRNOG00000011341	476.2863086	0.263473611	0.081931596	0.049100398
Mul1	ENSRNOG00000016010	356.2106623	0.265189431	0.071225119	0.017417348
Sacs	ENSRNOG00000014509	2604.299098	0.265631572	0.064395583	0.009024782
Dmxl2	ENSRNOG00000009170	3484.406509	0.266844114	0.068669238	0.014594676
Cdk5	ENSRNOG00000008017	709.3876326	0.270734288	0.081736575	0.03994529
Pls3	ENSRNOG00000027520	2276.667925	0.276149974	0.073392467	0.016643171
Tafa2	ENSRNOG00000004180	623.882981	0.276593477	0.073987462	0.017296157
Zfp365	ENSRNOG00000000638	2188.92904	0.27781165	0.086116887	0.048045175
Lzts1	ENSRNOG00000011826	558.7866461	0.278363385	0.074892984	0.017417348
Nek7	ENSRNOG00000000657	726.4878322	0.282886954	0.085402283	0.03994529
Fzd10	ENSRNOG00000022784	546.6868255	0.283732859	0.088401198	0.04934243
Setd7	ENSRNOG00000013045	879.2626664	0.284753551	0.080575712	0.025865101
Sh3bp4	ENSRNOG00000019316	707.22841	0.284829093	0.074683579	0.015874491
Insyn1	ENSRNOG00000026238	1490.857867	0.285288117	0.074077791	0.014988691
Clcn4	ENSRNOG00000003533	2027.535201	0.288049636	0.08064761	0.024047382
Atp8a2	ENSRNOG00000008053	398.6719665	0.288667043	0.086967909	0.039594758
Nefl	ENSRNOG00000013658	9291.026739	0.289520626	0.077293981	0.017122637
Gnal	ENSRNOG00000010440	896.1866089	0.290474025	0.076022358	0.015763733
Klhl11	ENSRNOG00000016921	717.7305142	0.293214892	0.080583468	0.020450794
Abhd8	ENSRNOG00000000054	1938.21155	0.293608035	0.085178682	0.031528797
Slc36a4	ENSRNOG00000011455	779.7040538	0.293725149	0.074774902	0.013876716
Wls	ENSRNOG00000036816	1297.307625	0.293780419	0.086212102	0.033572986
Pgk1	ENSRNOG00000058249	1555.454491	0.297901394	0.075526552	0.013876716
Tdrd7	ENSRNOG00000055779	753.2385363	0.298211309	0.09038968	0.040560641
Vcpip1	ENSRNOG00000006980	870.4553525	0.30052901	0.074756825	0.011833723
Dagla	ENSRNOG00000027264	1572.868581	0.301293976	0.090499878	0.038602826
Gpc1	ENSRNOG00000049437	1608.470545	0.303784421	0.088984551	0.033483408
Atxn7l3	ENSRNOG00000020930	884.9030417	0.305711596	0.091821346	0.038602826
Acbd5	ENSRNOG00000017642	296.4734813	0.30653887	0.082283838	0.017417348
Unc80	ENSRNOG00000028362	2261.779329	0.306755876	0.083023908	0.017954963
Cachd1	ENSRNOG00000010514	697.9434661	0.309976663	0.069893277	0.004063444
Stmn1	ENSRNOG00000016810	3714.454517	0.310288805	0.067725657	0.002389365
Psd3	ENSRNOG00000013884	687.2806127	0.310350448	0.094431633	0.041956653
Rasal2	ENSRNOG00000004917	445.6634117	0.31075396	0.092434967	0.036386727

Gene symbol	Ensembl gene	baseMean	log2FoldChange	lfcSE	*Padj*
Faxc	ENSRNOG00000010106	158.0245594	0.31107148	0.092450063	0.036345724
Bhlhe40	ENSRNOG00000007152	844.6807028	0.316886271	0.08615457	0.018486492
Ube2e3	ENSRNOG00000004544	760.2991826	0.320179881	0.08246734	0.014594676
Cx3cl1	ENSRNOG00000016326	4608.378992	0.320293004	0.090420089	0.025610565
Agfg1	ENSRNOG00000015619	362.6240544	0.321277722	0.094209101	0.033572986
Myo9a	ENSRNOG00000011619	820.8794678	0.321278337	0.094286442	0.033572986
Ctnnb1	ENSRNOG00000054172	3560.944348	0.321855374	0.078744811	0.009817929
Hecw1	ENSRNOG00000016046	533.4493791	0.325410214	0.069318171	0.001804382
Tes	ENSRNOG00000051952	218.7614549	0.326017532	0.098561433	0.040128774
Camk1d	ENSRNOG00000017882	939.5332303	0.32640986	0.095091025	0.032756232
Rorb	ENSRNOG00000013413	309.6686772	0.326745569	0.097260965	0.036579751
Palm2	ENSRNOG00000025558	235.5185325	0.328605896	0.097095527	0.035313523
Hspa12a	ENSRNOG00000018019	5988.32322	0.329996669	0.097428219	0.035082435
Prex2	ENSRNOG00000005391	1512.83793	0.331169964	0.070028694	0.001594929
Igsf21	ENSRNOG00000049959	375.2221636	0.331406105	0.089128052	0.017417348
Ctnna2	ENSRNOG00000006003	981.4164854	0.332175703	0.091493194	0.020727709
Enc1	ENSRNOG00000016541	4401.018262	0.333087933	0.091669049	0.020727709
Slc9a7	ENSRNOG00000004150	390.0289903	0.341449955	0.09401885	0.020727709
Etv1	ENSRNOG00000006867	1777.174967	0.342222811	0.080952548	0.007139075
Frrs1l	ENSRNOG00000043103	324.0531853	0.343228503	0.089820931	0.015763733
Lmbrd2	ENSRNOG00000054751	502.2481525	0.34330074	0.09353059	0.018724419
Grm1	ENSRNOG00000014290	1908.226329	0.345303304	0.082866599	0.00804097
Megf9	ENSRNOG00000005932	1573.563718	0.346404461	0.10466692	0.040128774
Cbln2	ENSRNOG00000013654	1320.963409	0.346638123	0.099179377	0.028038623
Ttll7	ENSRNOG00000031997	3318.861945	0.347869053	0.098239022	0.025618809
Rab3ip	ENSRNOG00000005362	345.4333643	0.348777668	0.093684388	0.017417348
Nudt4	ENSRNOG00000009094	4210.150761	0.349384406	0.102178755	0.033448273
Ppp2ca	ENSRNOG00000005389	243.6985782	0.349912072	0.09150411	0.015763733
Uhmk1	ENSRNOG00000002941	1250.873974	0.350036268	0.101302517	0.031247429
Rgs8	ENSRNOG00000002369	3854.145232	0.350125754	0.094351957	0.017714812
Mmd	ENSRNOG00000002436	2142.454331	0.351189062	0.091968366	0.015763733
Adam22	ENSRNOG00000042478	616.3721878	0.351501086	0.084005323	0.007585769
Slitrk6	ENSRNOG00000022337	1020.405141	0.35267883	0.109576709	0.049054084
Zyg11b	ENSRNOG00000010859	803.8174165	0.353539782	0.096641594	0.019238274
Tln2	ENSRNOG00000018373	1350.36383	0.354673855	0.093604307	0.016000214
Btbd3	ENSRNOG00000008088	2235.968049	0.356365931	0.104328354	0.033483408
Lmo1	ENSRNOG00000014629	181.7451196	0.357290862	0.109996623	0.046021784
Rims3	ENSRNOG00000011171	1185.654683	0.358389237	0.105725579	0.034902007
Cyp46a1	ENSRNOG00000007147	2056.708451	0.359522676	0.100811531	0.024218402
Rap1gds1	ENSRNOG00000015987	6162.908331	0.359896964	0.108898088	0.040292516
Pex5l	ENSRNOG00000011211	1279.680465	0.360223294	0.101901114	0.025865101
Ppm1e	ENSRNOG00000024730	1442.624307	0.360356501	0.09196388	0.014229119
Cacna2d3	ENSRNOG00000031287	1451.5598	0.361564277	0.10291974	0.027406325
Atxn1	ENSRNOG00000016998	445.7298963	0.361658845	0.107730158	0.036666364
Chrnb2	ENSRNOG00000020778	1290.528567	0.361984311	0.097364256	0.017417348
Slc36a1	ENSRNOG00000012356	933.8769121	0.363674164	0.099525134	0.01945385
C1qtnf4	ENSRNOG00000009140	581.6885093	0.367338808	0.096883766	0.016000214

Gene symbol	Ensembl gene	baseMean	log2FoldChange	lfcSE	*Padj*
Lrtm2	ENSRNOG00000007508	954.7480909	0.367384479	0.109590767	0.036953846
Dbndd1	ENSRNOG00000026974	695.3140269	0.36942146	0.107058962	0.031386543
Frmpd4	ENSRNOG00000004118	998.8122885	0.369845507	0.110621931	0.037938142
Scrt1	ENSRNOG00000025594	777.9504267	0.369858297	0.105810986	0.028038623
Ift57	ENSRNOG00000001958	613.95574	0.370706422	0.089839699	0.009024782
Pcsk2	ENSRNOG00000005438	5895.608194	0.371750038	0.107206658	0.030106439
Plekhd1	ENSRNOG00000038297	536.9216184	0.373868016	0.114243019	0.043476298
Tpd52l1	ENSRNOG00000021478	587.2757739	0.37470361	0.114370626	0.04327421
Hlf	ENSRNOG00000002456	555.5321977	0.375941958	0.096530267	0.014594676
Fam210b	ENSRNOG00000004466	417.1312994	0.385454693	0.098122136	0.013876716
Birc6	ENSRNOG00000027191	1025.24224	0.385880203	0.091209565	0.007139075
Slc17a6	ENSRNOG00000016147	8226.22125	0.387789574	0.100813986	0.014988691
Kcnj12	ENSRNOG00000002303	425.2160518	0.387949487	0.1051884	0.018131821
Sv2b	ENSRNOG00000011160	3094.292113	0.389065641	0.110085661	0.025865101
Tmem132d	ENSRNOG00000008447	182.4232743	0.390423145	0.103959272	0.016786991
Zmat4	ENSRNOG00000042690	1499.615887	0.39082942	0.116089069	0.036214224
Nhlh2	ENSRNOG00000054375	201.0106531	0.392661591	0.121116464	0.046623932
Vps13c	ENSRNOG00000030213	950.0397114	0.39451463	0.075236508	0.000253731
Cntn4	ENSRNOG00000005652	981.8459078	0.396574978	0.093794034	0.007139075
Plcxd2	ENSRNOG00000042289	684.4730097	0.39717203	0.099114323	0.011897901
Pdk3	ENSRNOG00000012513	302.4202675	0.397520235	0.093336659	0.007091237
Slc41a3	ENSRNOG00000045821	170.2793546	0.407307568	0.119411918	0.033572986
Syt7	ENSRNOG00000026432	2119.969868	0.410245776	0.119796263	0.033325221
Rnf152	ENSRNOG00000014859	826.3089946	0.412372385	0.124393459	0.039897156
Mcf2	ENSRNOG00000003435	237.7530469	0.414538545	0.128461537	0.048040998
Arfgef3	ENSRNOG00000011460	573.8296221	0.41501929	0.109678633	0.016000214
Inpp4b	ENSRNOG00000018382	527.9499988	0.415468641	0.129703376	0.04982643
Taok1	ENSRNOG00000015692	476.7977728	0.417301555	0.12102374	0.031515158
Sel1l3	ENSRNOG00000004932	611.3618827	0.417323644	0.124381612	0.036688213
Fam135b	ENSRNOG00000005159	170.0353135	0.420747334	0.127445311	0.040468902
Zdhhc22	ENSRNOG00000011285	960.6216583	0.421911416	0.127503152	0.040128774
Epha8	ENSRNOG00000013036	143.6576674	0.422289985	0.127125763	0.039404769
Fat3	ENSRNOG00000011585	504.3183016	0.423754009	0.098871208	0.006590968
Rab3c	ENSRNOG00000011623	1603.495121	0.423908806	0.111997843	0.016000214
Hdac9	ENSRNOG00000004158	322.5462952	0.424764558	0.120505158	0.026434759
Cacnb4	ENSRNOG00000007666	1420.294682	0.429794045	0.116171339	0.017809851
Galnt14	ENSRNOG00000007951	189.6676771	0.437943876	0.120901379	0.020944189
Ogfrl1	ENSRNOG00000014142	672.9820351	0.439119881	0.116009386	0.016000214
Slc8a1	ENSRNOG00000008479	575.5018949	0.439282412	0.09257761	0.001547919
Stxbp5l	ENSRNOG00000002496	216.4718376	0.441583072	0.104769751	0.007139075
Atp2b1	ENSRNOG00000004026	7693.592131	0.444859843	0.128936739	0.031386543
Epha4	ENSRNOG00000013213	3699.461648	0.446114763	0.112569157	0.013439116
Myo5a	ENSRNOG00000058866	4283.3543	0.446502231	0.096757333	0.00233865
Cndp2	ENSRNOG00000015591	1227.295408	0.44688912	0.089579112	0.000627615
Pcdhb19	ENSRNOG00000039469	108.5987029	0.450246247	0.113811528	0.013631238
Pamr1	ENSRNOG00000005348	1002.560596	0.456205467	0.127874262	0.024218402

Gene symbol	Ensembl gene	baseMean	log2FoldChange	lfcSE	*Padj*
Vav3	ENSRNOG00000020485	875.9139404	0.458462293	0.130899334	0.027945251
Reps2	ENSRNOG00000061508	354.9253593	0.458479639	0.121839773	0.016643171
Sprn	ENSRNOG00000018927	207.317064	0.45877415	0.137690669	0.038602826
Wnt3	ENSRNOG00000003845	215.5822974	0.460310368	0.142111687	0.046785136
Lyst	ENSRNOG00000058094	294.475479	0.460716867	0.111652709	0.009024782
Chst2	ENSRNOG00000047734	697.1774828	0.461707651	0.10919616	0.007139075
Mgll	ENSRNOG00000014508	1030.805628	0.463665866	0.124236055	0.017296157
Scn1b	ENSRNOG00000021102	2557.349417	0.464338764	0.121634995	0.015763733
Wnk3	ENSRNOG00000002537	224.3004118	0.464849094	0.125386971	0.017773634
Cpne9	ENSRNOG00000023077	954.3877739	0.465776317	0.119827115	0.014594676
Mb21d2	ENSRNOG00000024040	1174.174214	0.473167295	0.127230505	0.017417348
Cck	ENSRNOG00000019321	1667.652576	0.478947693	0.130258905	0.018486492
Tcf7l2	ENSRNOG00000049232	4399.174822	0.489024298	0.119104864	0.009347122
Dok6	ENSRNOG00000038190	205.0391255	0.49380021	0.114156586	0.006103747
Bok	ENSRNOG00000018214	829.9583299	0.494633078	0.134323485	0.018445223
Kdm7a	ENSRNOG00000052445	131.0223207	0.496145863	0.15222541	0.044825416
Gabrb1	ENSRNOG00000002327	248.1106806	0.501127724	0.138923276	0.021883712
Adarb2	ENSRNOG00000030775	296.9756259	0.502444509	0.131426657	0.015763733
Socs4	ENSRNOG00000011377	71.52968296	0.50283514	0.134586514	0.017296157
Pdp1	ENSRNOG00000016180	2728.034995	0.505111767	0.135889185	0.017417348
Syndig1l	ENSRNOG00000027645	315.7591218	0.512115678	0.144346236	0.025322089
Camk4	ENSRNOG00000020478	208.2828586	0.516115118	0.126080108	0.00970485
Vps13a	ENSRNOG00000025539	404.007429	0.517355666	0.122617915	0.007139075
Tp53i11	ENSRNOG00000008738	818.4093613	0.520551206	0.103841813	0.000612337
Ildr2	ENSRNOG00000025151	552.6827281	0.52132112	0.144665379	0.022083385
Necab3	ENSRNOG00000016708	876.9268937	0.523150796	0.123480358	0.007139075
Osbpl3	ENSRNOG00000010011	527.7394698	0.526459751	0.143219683	0.018486492
Atrnl1	ENSRNOG00000017406	1205.623621	0.533532093	0.124910705	0.006870484
Gabrb2	ENSRNOG00000003680	1379.920674	0.541306078	0.12211535	0.004063444
Itpr1	ENSRNOG00000007104	3867.757846	0.546511366	0.127125477	0.006399321
Aldoart2	ENSRNOG00000030869	99.61883598	0.550819222	0.141122295	0.014594676
Pmaip1	ENSRNOG00000018770	271.5835741	0.561196941	0.125361508	0.003632301
Kcnmb4	ENSRNOG00000054458	232.31266	0.562624237	0.173849764	0.046983522
Ago3	ENSRNOG00000034269	56.4487641	0.578289075	0.178445618	0.046715875
Fzd3	ENSRNOG00000047211	228.7564792	0.582211846	0.150139914	0.014627981
Trnp1	ENSRNOG00000055936	150.5240723	0.599069928	0.1493242	0.011897901
Scai	ENSRNOG00000025278	53.48191385	0.599356931	0.179888353	0.038602826
F13a1	ENSRNOG00000015957	189.7030354	0.613363797	0.176485242	0.029484032
Wnt9b	ENSRNOG00000003807	646.7922202	0.616903556	0.152225178	0.010901649
Kcnq3	ENSRNOG00000005206	400.2331286	0.623312865	0.14119666	0.004175993
Sema3d	ENSRNOG00000007202	277.5519749	0.627565262	0.134026354	0.001830512
Foxp2	ENSRNOG00000054508	803.8614382	0.636012791	0.109202054	4.26E-05
Cnih3	ENSRNOG00000022724	53.74365365	0.63797124	0.191074199	0.038265163
Gabrd	ENSRNOG00000016385	680.9541263	0.652840111	0.203775704	0.04982643
Xkr4	ENSRNOG00000027276	92.38978755	0.660845686	0.171139754	0.014988691
Epop	ENSRNOG00000048187	50.02133319	0.661013528	0.1786205	0.017809851
Disp3	ENSRNOG00000026447	154.9794987	0.673011605	0.15166393	0.004063444

Gene symbol	Ensembl gene	baseMean	log2FoldChange	lfcSE	*Padj*
Megf11	ENSRNOG00000010634	174.4524558	0.673276612	0.127630506	0.000246142
Wnt4	ENSRNOG00000013166	461.3861389	0.676250833	0.137671548	0.000787213
Lypd6	ENSRNOG00000038980	486.0666468	0.676868115	0.129456974	0.000253731
Ranbp2	ENSRNOG00000056428	47.31844126	0.677037346	0.186639304	0.020727709
Epha6	ENSRNOG00000029184	118.9208572	0.686249196	0.180602146	0.016000214
Mdfic	ENSRNOG00000053787	92.34536377	0.690358857	0.177898469	0.014594676
Wnt2b	ENSRNOG00000014385	82.96884217	0.704142222	0.185178548	0.016000214
Prkcq	ENSRNOG00000019057	532.9662267	0.712799918	0.120076138	4.26E-05
Lhx9	ENSRNOG00000010357	119.7428395	0.738036212	0.18934732	0.014594676
Ptar1	ENSRNOG00000014891	68.35082952	0.743873018	0.162097955	0.002389365
Tnc	ENSRNOG00000058645	433.0602998	0.749829306	0.224706121	0.038272127
Grin2a	ENSRNOG00000033942	294.2355249	0.752281593	0.151173426	0.000627615
Lnpep	ENSRNOG00000047387	80.97569214	0.773174976	0.179893835	0.006399321
Styk1	ENSRNOG00000010347	88.9665813	0.787307492	0.210554356	0.017296157
Lnpep	ENSRNOG00000055229	87.45976602	0.804364643	0.16022806	0.000612337
Rora	ENSRNOG00000027145	170.8501767	0.80744288	0.201064627	0.011886233
Cbln4	ENSRNOG00000004372	882.2620517	0.841227442	0.183692303	0.002389365
Kcnh7	ENSRNOG00000007528	56.13176725	0.897583562	0.169909535	0.000246142
Zfp871	ENSRNOG00000004947	92.79826377	0.900149715	0.156768783	4.63E-05
Oprm1	ENSRNOG00000018191	187.0866108	0.965136599	0.243576544	0.013439116
Grifin	ENSRNOG00000001251	25.52891709	0.999486315	0.287597759	0.029484032
Lypd6b	ENSRNOG00000004614	245.6907108	1.001501824	0.237454257	0.007139075
Lrp1b	ENSRNOG00000030243	25.96291413	1.0082661	0.256711263	0.013876716
Fut9	ENSRNOG00000008475	46.37577146	1.028441575	0.267521441	0.014988691
Ctxn3	ENSRNOG00000022957	248.7995159	1.048279145	0.305013449	0.032489309
Kcnh5	ENSRNOG00000009542	82.70350855	1.070396872	0.193204158	0.000112133
Fzd5	ENSRNOG00000014678	44.87895786	1.137445729	0.210269323	0.00016489
Acpp	ENSRNOG00000011820	30.04738876	1.228734894	0.285102503	0.006385145
NA	ENSRNOG00000051264	29.76124212	-1.089465025	0.304708654	0.023926068
NA	ENSRNOG00000005953	33.25227526	-0.878602486	0.237624286	0.017866485
NA	ENSRNOG00000048751	65.32734856	-0.642519513	0.199123001	0.048040998
NA	ENSRNOG00000062102	280.761364	-0.555748595	0.138833551	0.011907436
NA	ENSRNOG00000049150	122.6776106	-0.517600726	0.128377729	0.011412633
NA	ENSRNOG00000057903	54.84695712	-0.509083584	0.155933274	0.04432838
NA	ENSRNOG00000052540	99.96968229	-0.507836443	0.143610018	0.025865101
NA	ENSRNOG00000062181	411.0759735	-0.474914073	0.126291036	0.016660598
NA	ENSRNOG00000061630	220.4705484	-0.360581648	0.085784471	0.007232022
NA	ENSRNOG00000053620	985.2507741	0.295328183	0.09094774	0.046021784
NA	ENSRNOG00000056731	652.0348917	0.298867568	0.074600801	0.011897901
NA	ENSRNOG00000054121	381.9155876	0.308557847	0.08337598	0.017809851
NA	ENSRNOG00000048800	293.4544258	0.318587976	0.081709091	0.014594676
NA	ENSRNOG00000057161	200.122593	0.464742819	0.13279688	0.028038623
NA	ENSRNOG00000059113	344.7742146	0.455633487	0.112113132	0.010531496
NA	ENSRNOG00000053968	179.7681396	0.536685483	0.14188061	0.016000214
NA	ENSRNOG00000060482	34.77113027	0.699939141	0.210154903	0.038602826
NA	ENSRNOG00000012103	68.22109562	0.801028174	0.192829529	0.008361707

Gene symbol	Ensembl gene	baseMean	log2FoldChange	lfcSE	*Padj*
NA	ENSRNOG00000059746	38.1621653	0.810706512	0.225507702	0.022612075
LOC100910996	ENSRNOG00000060879	141.9359654	-0.521100963	0.147606608	0.026003404
RGD1304884	ENSRNOG00000017431	3095.958522	0.17064831	0.049675145	0.032553937

1 vs. 3 Loud noise exposures

Gene symbol	Ensembl gene	baseMean	log2FoldChange	lfcSE	*Padj*
Ubash3b	ENSRNOG00000008187	487.9694556	-0.5053916	0.1112114	0.0444097
Kcnh5	ENSRNOG00000009542	82.70350855	1.0383397	0.2039368	0.0064907
Zfp871	ENSRNOG00000004947	92.79826377	0.7094682	0.1581882	0.0444097

1 vs. 7 Loud noise exposures

Gene symbol	Ensembl gene	baseMean	log2FoldChange	lfcSE	*Padj*
Avp	ENSRNOG00000021229	274.385853	-4.180099552	1.196785035	0.02125918
Oxt	ENSRNOG00000021225	381.4672397	-3.76512601	1.18382689	0.042230992
Trh	ENSRNOG00000011824	393.1827108	-2.148230722	0.606220041	0.018662026
Prlhr	ENSRNOG00000009922	24.58690572	-2.117719038	0.574423005	0.012700884
Kcnj4	ENSRNOG00000013869	23.22839264	-1.766708621	0.384495294	0.001089777
Isl1	ENSRNOG00000012556	99.95575544	-1.456505696	0.388228527	0.010597537
Ntrk1	ENSRNOG00000013953	20.93042367	-1.347740374	0.334930616	0.005175934
Myo15b	ENSRNOG00000042445	24.12863767	-1.153452284	0.349406158	0.033247748
Gck	ENSRNOG00000061527	40.69867367	-1.015293898	0.257168405	0.00635003
Esyt3	ENSRNOG00000022704	63.20203089	-0.979663968	0.260771463	0.010473773
Crhr2	ENSRNOG00000011145	34.70884024	-0.925757079	0.27716442	0.029965644
Trpc7	ENSRNOG00000012727	54.87369557	-0.860573293	0.205828639	0.003693286
Daw1	ENSRNOG00000016247	24.44828507	-0.85818463	0.234169933	0.013127443
Sim1	ENSRNOG00000037600	113.8388682	-0.856127492	0.213999981	0.005372388
Btc	ENSRNOG00000002728	26.75539512	-0.850057914	0.210480197	0.005020406
Frem3	ENSRNOG00000039152	81.89705398	-0.820842192	0.254577236	0.038658153
Scn5a	ENSRNOG00000015049	130.910339	-0.817411943	0.200096928	0.004512217
Tgfbi	ENSRNOG00000012216	76.76308475	-0.815573197	0.195115073	0.003693286
Nppa	ENSRNOG00000008176	32.04759731	-0.788794092	0.247837516	0.042150762
Myh7	ENSRNOG00000016983	28.30903191	-0.78171924	0.248999411	0.045610801
Ndnf	ENSRNOG00000006857	106.9303805	-0.76436299	0.232977352	0.034462588
Adamtsl4	ENSRNOG00000049385	31.16080098	-0.764350502	0.228503966	0.029752249
Lbhd2	ENSRNOG00000043137	23.01057912	-0.755054405	0.240948177	0.046186862
Islr2	ENSRNOG00000050714	128.0832703	-0.749393953	0.210246682	0.017699964
RT1-A2	ENSRNOG00000030712	126.3239429	-0.743569494	0.207250947	0.01650781
Arhgap36	ENSRNOG00000007552	367.5291032	-0.73577014	0.218751195	0.02886135
Arc	ENSRNOG00000043465	68.10172022	-0.716111966	0.183139259	0.007014693
Penk	ENSRNOG00000008943	595.6618097	-0.707026278	0.225032583	0.045485769
Cfap300	ENSRNOG00000043410	75.56096076	-0.701845146	0.196107582	0.016855191
Gad1	ENSRNOG00000000007	2773.191849	-0.701027039	0.124035111	1.54E-05
Wscd2	ENSRNOG00000053045	78.41157543	-0.696151799	0.159285722	0.002068605
Icam5	ENSRNOG00000020694	93.1197978	-0.688801848	0.210776104	0.035433711

Gene symbol	Ensembl gene	baseMean	log2FoldChang	lfcSE	*Padj*
Dmc1	ENSRNOG00000013807	55.79145129	-0.677936827	0.196064091	0.02331103
Actn2	ENSRNOG00000017833	68.23843326	-0.674303632	0.157437033	0.002762785
Cdhr1	ENSRNOG00000013330	56.64479556	-0.671179426	0.180446542	0.01153139
Cldn9	ENSRNOG00000003654	33.28036637	-0.665641822	0.205368392	0.037208157
Il22ra2	ENSRNOG00000012259	44.6502258	-0.665580693	0.186805869	0.017736824
Tmem215	ENSRNOG00000042246	62.95692251	-0.664565927	0.211813024	0.045669584
Arx	ENSRNOG00000053562	121.0290492	-0.660247027	0.196744818	0.02929812
Ubxn10	ENSRNOG00000027731	35.85628892	-0.652489183	0.207881649	0.045610801
Fam20a	ENSRNOG00000003969	61.19431207	-0.635392101	0.181634042	0.021077064
Magel2	ENSRNOG00000010158	324.0697689	-0.613101197	0.144027373	0.003048286
Zbtb7c	ENSRNOG00000047924	76.65543216	-0.611624501	0.150240795	0.00466209
Prss23	ENSRNOG00000017307	112.1214722	-0.606846625	0.191701953	0.043445969
Pla2g4b	ENSRNOG00000007447	70.75185992	-0.60335995	0.158347629	0.009452272
Zdhhc23	ENSRNOG00000060348	130.7564688	-0.5994423	0.148728246	0.005119018
Slc44a5	ENSRNOG00000042332	62.48954883	-0.59375184	0.161277206	0.012748718
Ubash3b	ENSRNOG00000008187	487.9694556	-0.573592538	0.111040033	0.000127076
Baiap3	ENSRNOG00000017893	1759.756359	-0.558460631	0.122608468	0.001179554
Gucy1a1	ENSRNOG00000012302	440.6497415	-0.546269656	0.108279368	0.000180303
Gad2	ENSRNOG00000018200	4964.640114	-0.543313494	0.131142679	0.003916859
Fgl2	ENSRNOG00000012881	154.5396811	-0.54093605	0.140808487	0.008600675
Scml4	ENSRNOG00000026110	83.44308795	-0.54015482	0.161086027	0.029362023
Kiss1r	ENSRNOG00000011954	60.55563898	-0.53503681	0.17251799	0.049555614
Spsb4	ENSRNOG00000012862	48.82118168	-0.528234523	0.167451935	0.044313506
Stom	ENSRNOG00000019147	136.8544261	-0.527477992	0.124729547	0.003227728
Ngef	ENSRNOG00000016653	401.5762393	-0.521607282	0.128559851	0.004850082
Afmid	ENSRNOG00000050205	137.8852849	-0.517650652	0.134039836	0.007989083
Dysf	ENSRNOG00000032788	60.77879481	-0.517647132	0.146587683	0.019308398
Rxrg	ENSRNOG00000004537	108.8990897	-0.515160806	0.162179734	0.042324952
Catip	ENSRNOG00000037835	77.08164133	-0.51505493	0.154363745	0.030269225
Cdh9	ENSRNOG00000033837	100.5838786	-0.513188449	0.152181403	0.028548818
Slc32a1	ENSRNOG00000015393	1070.89168	-0.510888838	0.12654885	0.005022561
Celf6	ENSRNOG00000052224	378.3416167	-0.501075262	0.110773415	0.001272556
Crocc	ENSRNOG00000008334	226.0387012	-0.498503176	0.126365575	0.006372596
Irf1	ENSRNOG00000008144	67.73920486	-0.49575842	0.144517237	0.024935242
Rerg	ENSRNOG00000027592	151.204491	-0.489355364	0.153750258	0.042150762
Fbxl6	ENSRNOG00000025497	81.60759008	-0.489072626	0.139986185	0.02125918
Hap1	ENSRNOG00000014819	4275.232858	-0.4889815	0.122872529	0.005663628
Spint2	ENSRNOG00000020636	249.6480782	-0.488883192	0.125996688	0.00763676
Hspb8	ENSRNOG00000022392	200.1523762	-0.480142508	0.103776403	0.000977133
Slc27a3	ENSRNOG00000015421	161.7201517	-0.473201515	0.121035945	0.007014693
Mgst1	ENSRNOG00000007743	150.8399705	-0.459495878	0.147502921	0.048487994
Fchsd1	ENSRNOG00000039415	134.8557917	-0.452130347	0.125775912	0.016301681
Slit2	ENSRNOG00000003840	350.7592095	-0.450348981	0.14482033	0.048902275
Pla2g2c	ENSRNOG00000016647	107.9363163	-0.449405154	0.12928023	0.022441416

Gene symbol	Ensembl gene	baseMean	log2FoldChang	lfcSE	*Padj*
Matn2	ENSRNOG00000006060	129.2645515	-0.449084739	0.13035211	0.023939083
Tmem203	ENSRNOG00000010894	74.76648677	-0.435362065	0.133820318	0.036189591
Qpct	ENSRNOG00000005705	232.2490003	-0.42860418	0.137896925	0.04897967
P3h3	ENSRNOG00000016071	464.1275844	-0.414850151	0.109758369	0.010007962
Npr2	ENSRNOG00000015991	568.1890562	-0.408684902	0.094751456	0.002541485
Zfpm2	ENSRNOG00000004109	143.2607508	-0.397371709	0.121465601	0.035147434
Il4r	ENSRNOG00000015441	143.3327483	-0.393482909	0.106776244	0.012700884
Ly6h	ENSRNOG00000007334	838.3747201	-0.386030665	0.106223992	0.014331686
Pnma3	ENSRNOG00000052022	747.0376837	-0.38429731	0.113742438	0.028304517
Hcn3	ENSRNOG00000020444	203.0362906	-0.383271193	0.085505674	0.001423305
Ndn	ENSRNOG00000010146	3266.694546	-0.379508834	0.095298523	0.005629994
Tmem196	ENSRNOG00000037435	420.6124883	-0.368008767	0.112753831	0.035559654
Trim34	ENSRNOG00000042686	97.40031145	-0.363774454	0.114261688	0.042150762
Kif20a	ENSRNOG00000024428	154.3389749	-0.36171036	0.109734307	0.033665179
Ptpro	ENSRNOG00000006231	651.5152194	-0.361172015	0.105668961	0.025527723
Pak6	ENSRNOG00000007925	270.8952644	-0.360203482	0.104087578	0.023242079
Cygb	ENSRNOG00000011541	687.0824608	-0.356351613	0.112278115	0.042550712
Ass1	ENSRNOG00000008837	369.5201898	-0.353319705	0.113313459	0.048258418
Anxa7	ENSRNOG00000007136	233.1177699	-0.353135589	0.090390524	0.007014693
Tmem132e	ENSRNOG00000007455	244.008787	-0.350446066	0.105095819	0.030424794
As3mt	ENSRNOG00000020081	153.0656203	-0.346111107	0.108175298	0.0408056
Sema6c	ENSRNOG00000021101	178.1091714	-0.345566179	0.100295505	0.023939083
Nbeal2	ENSRNOG00000027880	214.1213976	-0.33812234	0.10100219	0.029731932
Pitpnm3	ENSRNOG00000008323	821.4715725	-0.336939702	0.076711048	0.001982446
Wdr6	ENSRNOG00000020185	2828.331755	-0.331926161	0.078435404	0.003216629
Gstm4	ENSRNOG00000019221	179.7405462	-0.330434688	0.101749836	0.036546511
Ssh3	ENSRNOG00000018878	488.4211533	-0.327755396	0.077229267	0.003153558
Myo16	ENSRNOG00000016483	307.9017809	-0.322344783	0.094460492	0.025967597
Nynrin	ENSRNOG00000048431	278.5380353	-0.321219998	0.099592508	0.038601825
Tmed3	ENSRNOG00000013889	249.4699361	-0.319501957	0.079999277	0.005485678
Trafd1	ENSRNOG00000001351	700.0028894	-0.317806428	0.080871808	0.006716194
Cpne6	ENSRNOG00000018399	535.680786	-0.317352997	0.100463134	0.044139831
Vps9d1	ENSRNOG00000028904	357.2236106	-0.316449899	0.076887782	0.004180374
P4ha2	ENSRNOG00000033663	208.5989273	-0.31436589	0.08490681	0.012146651
Ece2	ENSRNOG00000001715	897.0763258	-0.313966564	0.077923145	0.005119018
Dchs1	ENSRNOG00000031643	415.2274375	-0.309610868	0.08950086	0.023242079
Gaa	ENSRNOG00000047656	4515.680498	-0.307470842	0.093293866	0.033665179
Ampd2	ENSRNOG00000019240	796.3431685	-0.306063304	0.091252838	0.029362023
Vat1	ENSRNOG00000020684	1502.19611	-0.30410079	0.087798047	0.023206247
Trnau1ap	ENSRNOG00000055344	285.2444501	-0.299111372	0.067310967	0.001601065
Sgsm1	ENSRNOG00000000708	1772.345909	-0.29834294	0.076389957	0.00701565
Trim39	ENSRNOG00000000785	321.8112379	-0.296209651	0.075263277	0.006588591
NA	ENSRNOG00000053537	595.5892341	-0.2953321	0.08041609	0.012873188
Eml3	ENSRNOG00000019873	237.3488742	-0.293589158	0.092992418	0.044139831
Slc7a3	ENSRNOG00000004133	368.8323528	-0.292771393	0.093146206	0.045457234

Gene symbol	Ensembl gene	baseMean	log2FoldChange	lfcSE	*Padj*
Gprasp2	ENSRNOG00000037658	4308.60741	-0.29151423	0.076423232	0.009338772
Klhdc8b	ENSRNOG00000047867	265.8729525	-0.291119738	0.082143637	0.018662026
Tmem19	ENSRNOG00000003985	200.7969322	-0.286730376	0.088247422	0.036412954
Psd	ENSRNOG00000019435	661.8742588	-0.286255596	0.073577045	0.007352084
Pir	ENSRNOG00000003674	157.1875803	-0.28551568	0.090974361	0.045610801
Ctbp2	ENSRNOG00000017326	332.7078269	-0.282852096	0.076760918	0.012700884
Ntrk3	ENSRNOG00000018674	406.8922875	-0.280350584	0.085627427	0.034980489
Itih3	ENSRNOG00000017689	10681.38075	-0.276691792	0.066180826	0.003693286
Tbl3	ENSRNOG00000013429	176.7684302	-0.273869864	0.087962767	0.048593878
Plppr3	ENSRNOG00000027940	683.3106797	-0.272545026	0.06974358	0.007014693
Col4a2	ENSRNOG00000023972	606.0348174	-0.271869118	0.086417421	0.045263632
Snx11	ENSRNOG00000008642	250.9091236	-0.269781454	0.083468089	0.038087275
Neurl1	ENSRNOG00000020339	716.3967291	-0.26799933	0.071616874	0.010881535
Cotl1	ENSRNOG00000016257	493.085032	-0.264351413	0.06823565	0.007752013
Prmt2	ENSRNOG00000001297	695.273222	-0.261905999	0.080470361	0.036094389
Cacna1i	ENSRNOG00000060407	493.1150303	-0.261686396	0.077079276	0.027172193
Igsf1	ENSRNOG00000007600	2767.234548	-0.260899149	0.077111033	0.027974187
Rab9b	ENSRNOG00000047960	1637.410581	-0.253356692	0.07751283	0.035430949
Irf3	ENSRNOG00000043388	451.7581155	-0.251885937	0.076401901	0.033665179
Rdh13	ENSRNOG00000027919	256.2937346	-0.248052961	0.077717368	0.041338328
Tmem63c	ENSRNOG00000011334	623.6891518	-0.24550668	0.061375108	0.005372388
Akr1b1-ps2	ENSRNOG00000023285	404.8136758	-0.245226575	0.066714186	0.012822556
Tmem130	ENSRNOG00000025235	11347.51525	-0.241322598	0.076580813	0.044627372
Glt8d1	ENSRNOG00000018179	331.1136906	-0.237228291	0.076105733	0.048258418
Agrn	ENSRNOG00000020205	1848.059523	-0.227272765	0.070008709	0.036619725
Parp8	ENSRNOG00000010824	358.443404	-0.22233251	0.070218026	0.043417181
Pgrmc1	ENSRNOG00000012786	3271.109515	-0.222250577	0.067550919	0.033777464
Jag2	ENSRNOG00000013927	522.130174	-0.221030301	0.068024546	0.036412954
Stk38	ENSRNOG00000000519	525.3055104	-0.219934066	0.057963509	0.009648773
Slc35f1	ENSRNOG00000000412	1232.655508	-0.21140401	0.068051374	0.04897967
Iqsec3	ENSRNOG00000014083	2238.608215	-0.20759131	0.052953598	0.006841166
Isyna1	ENSRNOG00000019741	450.9872441	-0.207242153	0.063209274	0.034634822
Slx4	ENSRNOG00000024445	501.326806	-0.206733296	0.058496425	0.019164937
MGC109340	ENSRNOG00000046858	1190.582637	-0.203671199	0.053774077	0.009780363
Zmym3	ENSRNOG00000003707	1556.896473	-0.19616271	0.062092936	0.044139831
Pitpnm2	ENSRNOG00000029260	1190.245993	-0.186042162	0.050594103	0.012822556
Impact	ENSRNOG00000045844	9931.962666	-0.185860488	0.059771362	0.048902275
Hnrnpa1	ENSRNOG00000036839	1229.544873	-0.180513797	0.053681057	0.02886135
Tra2a	ENSRNOG00000009156	853.9686683	-0.176482708	0.055159699	0.0408056
Ergic3	ENSRNOG00000031085	725.4262583	-0.175278312	0.055684311	0.045081875
Ddx39b	ENSRNOG00000000841	2682.345211	-0.174326494	0.051479968	0.027755682
Pip5k1a	ENSRNOG00000021068	694.6709693	-0.172821957	0.049920551	0.023217105
Dgkq	ENSRNOG00000024112	915.5540326	-0.172641239	0.051410177	0.029132334
Epdr1	ENSRNOG00000060141	1301.051442	-0.169298009	0.047133284	0.016357655

Gene symbol	Ensembl gene	baseMean	log2FoldChang	lfcSE	*Padj*
Gba2	ENSRNOG00000016364	1027.681239	-0.156620356	0.045799616	0.025435284
Tsc2	ENSRNOG00000011375	1313.617627	-0.155275012	0.047299895	0.034454339
Srsf2	ENSRNOG00000000248	2031.665089	-0.152310754	0.04832954	0.044627372
Tp53bp1	ENSRNOG00000013837	2401.441607	-0.138697268	0.043532527	0.042074424
Slc1a3	ENSRNOG00000016163	9215.785698	-0.124745414	0.039256584	0.042276382
Ube4b	ENSRNOG00000014986	1484.922667	0.120696171	0.037726875	0.0408056
Zfp91	ENSRNOG00000012524	3199.782811	0.132102215	0.032838506	0.005175934
Rcan2	ENSRNOG00000010350	5017.215017	0.13397627	0.038694036	0.023217105
Dixdc1	ENSRNOG00000010260	2252.018631	0.135638799	0.042095583	0.038860589
Hectd1	ENSRNOG00000006905	2600.411911	0.136083997	0.041309123	0.033760134
Arhgef12	ENSRNOG00000008924	1110.382888	0.136552402	0.041593227	0.034454339
Srcin1	ENSRNOG00000011475	1596.364001	0.137918726	0.041908803	0.033777464
Senp6	ENSRNOG00000024336	1320.521247	0.139439536	0.044101519	0.043932057
Agap1	ENSRNOG00000019476	2176.035483	0.141908949	0.04225503	0.029132334
Fam168b	ENSRNOG00000023467	2349.302549	0.142487709	0.043430697	0.034462588
Ctnnd1	ENSRNOG00000030790	1656.89446	0.146595834	0.046430012	0.044139831
Asrgl1	ENSRNOG00000020202	1596.12451	0.148468347	0.043515835	0.025967597
Prickle2	ENSRNOG00000012364	1869.356603	0.148742123	0.045776349	0.036412954
Cntn1	ENSRNOG00000004438	6567.465909	0.149111244	0.046572106	0.040732069
Camsap2	ENSRNOG00000008741	3967.830812	0.149807336	0.041033793	0.013571564
Secisbp2l	ENSRNOG00000008629	5257.016551	0.150361577	0.046605636	0.038558944
Dst	ENSRNOG00000012207	10009.90054	0.152095049	0.046004801	0.032923357
Nsf	ENSRNOG00000003905	11908.98892	0.157806737	0.045662249	0.023397597
Clasp1	ENSRNOG00000002376	2240.17196	0.157838734	0.045619958	0.023242079
Ddhd1	ENSRNOG00000009481	1180.230036	0.159617186	0.048488282	0.033777464
Macf1	ENSRNOG00000016047	7574.645374	0.167200048	0.042219184	0.006074296
Akap11	ENSRNOG00000009987	4012.864465	0.167694038	0.047318582	0.018662026
Cyfip2	ENSRNOG00000006557	11925.06866	0.16850954	0.054240799	0.04897967
Baalc	ENSRNOG00000004697	1740.185448	0.169247375	0.048059834	0.019659132
Jmjd1c	ENSRNOG00000000648	1375.723534	0.169845951	0.042368322	0.00537028
Camta2	ENSRNOG00000004283	2419.594266	0.170506173	0.050848947	0.029362023
Srpk2	ENSRNOG00000010601	4168.563537	0.171247933	0.054471391	0.045435563
Opa1	ENSRNOG00000001717	1657.561893	0.172071764	0.044561089	0.007989083
Ranbp2	ENSRNOG00000000796	2050.71601	0.172132254	0.050113139	0.024727428
Setx	ENSRNOG00000013491	987.3754351	0.173862361	0.053036938	0.034634822
Pxk	ENSRNOG00000008024	837.8443859	0.173937982	0.052836764	0.033777464
Myh10	ENSRNOG00000002886	5717.640601	0.175702873	0.04617234	0.00947744
Sbno1	ENSRNOG00000001064	1431.294077	0.17633777	0.049952675	0.019308602
Dpp8	ENSRNOG00000019105	1866.91512	0.178331682	0.039204163	0.001195767
Fam168a	ENSRNOG00000018873	3095.386035	0.181908573	0.04781019	0.00947744
Zfp106	ENSRNOG00000052583	3611.106826	0.182694385	0.048574532	0.010362983
Eps15	ENSRNOG00000010299	2191.365716	0.183434148	0.043744969	0.003645571
Ankrd40	ENSRNOG00000002935	2041.147585	0.184885579	0.041076596	0.001357197
Adam23	ENSRNOG00000012424	1351.981765	0.184993668	0.050506822	0.013185794
Ubr3	ENSRNOG00000008616	1639.042047	0.185148028	0.055359329	0.029752249

Gene symbol	Ensembl gene	baseMean	log2FoldChange	lfcSE	*Padj*
Neo1	ENSRNOG00000006490	1773.046583	0.187381383	0.051268583	0.01340502
Spata2	ENSRNOG00000009207	433.7759978	0.191259412	0.059279404	0.038558944
Nf1	ENSRNOG00000013780	1881.224044	0.191686225	0.053300063	0.016298589
Gcc2	ENSRNOG00000000823	1283.056939	0.192953276	0.059892505	0.038860589
Tnks	ENSRNOG00000011625	1017.504634	0.194271148	0.062383035	0.048566094
Abi2	ENSRNOG00000017707	1231.514428	0.194596981	0.05134191	0.009717425
Nmnat2	ENSRNOG00000027697	808.7287321	0.197236912	0.061500831	0.040554582
Mmp15	ENSRNOG00000012622	784.3046678	0.202500637	0.06516847	0.04897967
Tmf1	ENSRNOG00000056462	533.0043628	0.202824372	0.063035229	0.039248759
Wapl	ENSRNOG00000052513	676.439319	0.204218921	0.053756959	0.00954818
Fmn2	ENSRNOG00000061764	465.2292921	0.204721042	0.064134735	0.041338328
Adcy9	ENSRNOG00000049768	392.0697785	0.205265175	0.066087591	0.04897967
Myt1l	ENSRNOG00000004269	1615.978958	0.205550873	0.061471743	0.029752249
Sptbn2	ENSRNOG00000058842	4045.212088	0.206630045	0.059151027	0.02125918
Atrx	ENSRNOG00000056703	2117.79166	0.206854387	0.048801177	0.003153558
Pbx1	ENSRNOG00000004693	756.0278758	0.206935271	0.051370944	0.005119018
Acap2	ENSRNOG00000001730	572.6714592	0.210076217	0.064887568	0.03747517
Zbtb18	ENSRNOG00000004423	479.1302996	0.212095834	0.060554074	0.020796069
Nacc2	ENSRNOG00000018231	1709.999572	0.213451072	0.066117861	0.038437603
Stmn4	ENSRNOG00000053334	3612.962546	0.214197234	0.060792801	0.019586475
Uhrf1bp1l	ENSRNOG00000050317	1573.87998	0.214627632	0.059041888	0.014324551
Naa25	ENSRNOG00000001350	397.6301911	0.215339172	0.067723073	0.042261378
Epb41l3	ENSRNOG00000016724	4593.614446	0.21648991	0.059892677	0.015295286
Zfyve9	ENSRNOG00000027183	1445.84476	0.217309769	0.064635501	0.02886135
Ina	ENSRNOG00000020248	5770.622914	0.217382993	0.070127442	0.049659058
Dmxl2	ENSRNOG00000009170	3484.406509	0.217516801	0.068496574	0.042374593
Adcy8	ENSRNOG00000004890	765.4899399	0.219774189	0.069298958	0.042746541
Spock1	ENSRNOG00000012747	3048.303555	0.220809032	0.061428907	0.016301681
Vsnl1	ENSRNOG00000005345	7693.908656	0.222900184	0.062191773	0.016632385
Cdk14	ENSRNOG00000007151	3979.519312	0.222995782	0.070662189	0.04425893
Arap2	ENSRNOG00000056826	1431.568346	0.223241598	0.06035987	0.012291152
Prkcb	ENSRNOG00000012061	1599.431495	0.223249098	0.058857264	0.009648773
Plxdc2	ENSRNOG00000000142	556.7488227	0.225264823	0.065499427	0.024405172
Cpeb4	ENSRNOG00000033169	1069.709776	0.225678181	0.055212591	0.004497581
Trim37	ENSRNOG00000006248	2208.743527	0.225975845	0.057618286	0.006831726
Fryl	ENSRNOG00000002248	1653.310595	0.22699879	0.073202811	0.049555614
Napb	ENSRNOG00000004753	1505.138774	0.227524849	0.056775751	0.00537028
Thy1	ENSRNOG00000006604	10334.06958	0.228006173	0.070396484	0.037433708
Ankrd12	ENSRNOG00000012733	1673.740651	0.228165362	0.067672038	0.028548818
Oxr1	ENSRNOG00000056487	6024.225062	0.228754864	0.062080562	0.012700884
Ccdc88a	ENSRNOG00000004057	1059.060917	0.22881921	0.063050535	0.014560579
Pcdhgc3	ENSRNOG00000019799	2311.852977	0.229626979	0.067619068	0.027172193
Bod1l1	ENSRNOG00000050437	1305.186725	0.229831772	0.073861346	0.048806362
Ralgapa1	ENSRNOG00000046256	1589.596998	0.229991003	0.055221212	0.003730029

Gene symbol	Ensembl gene	baseMean	log2FoldChange	lfcSE	*Padj*
Lrrn1	ENSRNOG00000006802	1740.138625	0.230607415	0.070101107	0.033777464
Ank2	ENSRNOG00000011076	10162.88212	0.232314108	0.063219071	0.012822556
Trim9	ENSRNOG00000007031	1571.471549	0.232711011	0.074106438	0.04557757
Gdf11	ENSRNOG00000007610	1416.195414	0.232916085	0.056907149	0.004423394
Pak1	ENSRNOG00000029784	3901.47977	0.233178468	0.07062997	0.033247748
Wwp1	ENSRNOG00000006328	536.8296586	0.233846946	0.058623482	0.005522386
Cdh8	ENSRNOG00000056643	1174.371839	0.234415226	0.06194473	0.00986716
Fgf13	ENSRNOG00000042753	1814.704467	0.235548093	0.062001523	0.00954818
Usp15	ENSRNOG00000023202	727.7561593	0.236190056	0.062800139	0.010362983
Arpp21	ENSRNOG00000008919	838.2983048	0.237706824	0.066855268	0.018185123
Zbtb38	ENSRNOG00000012386	583.8321487	0.237862684	0.071142135	0.029752249
Kmt2c	ENSRNOG00000061080	646.0111138	0.23790707	0.063121483	0.010215569
Tut7	ENSRNOG00000016629	566.744939	0.23845475	0.056811293	0.003613271
Slc24a3	ENSRNOG00000060687	1566.398839	0.239188329	0.068012429	0.019953814
Ash1l	ENSRNOG00000020386	2732.809693	0.240638772	0.057684597	0.003693286
Septin5	ENSRNOG00000029912	3350.18487	0.240716062	0.071165977	0.027998654
Arhgap5	ENSRNOG00000004696	2064.780471	0.241040128	0.05775736	0.003693286
Arhgap32	ENSRNOG00000008709	4234.172117	0.241202358	0.075449052	0.040928336
Cdc42bpa	ENSRNOG00000002841	3015.451136	0.241697082	0.058007621	0.003730029
Gpr158	ENSRNOG00000024832	1456.354533	0.244482261	0.069318005	0.019444806
Zbtb7b	ENSRNOG00000020640	276.6026398	0.245769524	0.074342898	0.032923357
Dennd5b	ENSRNOG00000049378	577.3574086	0.246341243	0.074158064	0.031392078
Nedd4l	ENSRNOG00000017610	727.9983116	0.246647644	0.060607422	0.00466209
Smg1	ENSRNOG00000047386	1280.398495	0.246837282	0.061667144	0.005372388
Anks1b	ENSRNOG00000024870	1576.591971	0.248676571	0.069311987	0.01650781
Ccdc186	ENSRNOG00000051680	1000.186606	0.250268837	0.068078348	0.012822556
Kcnab2	ENSRNOG00000011550	1369.587677	0.250499413	0.073586212	0.026563018
Syncrip	ENSRNOG00000000204	338.5784093	0.251052026	0.078586659	0.041174491
Snap25	ENSRNOG00000006037	19590.50234	0.25108719	0.056194332	0.001482695
Wwc1	ENSRNOG00000008065	1080.59409	0.253022232	0.055501716	0.001175179
Fgf12	ENSRNOG00000001931	1177.632633	0.253435018	0.060317545	0.00357647
Med13	ENSRNOG00000003679	738.3848541	0.256111842	0.063262412	0.004940208
Arhgap44	ENSRNOG00000003603	1810.466929	0.257376276	0.076879275	0.029731932
Dmxl1	ENSRNOG00000024671	726.4538926	0.257524293	0.066125304	0.00726979
Dnajb14	ENSRNOG00000060107	377.7307166	0.259201322	0.07085272	0.013308656
Dlc1	ENSRNOG00000010780	1125.661311	0.259691335	0.079653006	0.035695693
Apba1	ENSRNOG00000014928	2168.076825	0.26052476	0.07392341	0.019584051
Rims2	ENSRNOG00000004201	1747.281707	0.26148569	0.084247097	0.049253278
Nefl	ENSRNOG00000013658	9291.026739	0.262079944	0.077239949	0.027204931
Adgrl2	ENSRNOG00000032660	1161.569561	0.263933528	0.064829714	0.00466209
Pds5a	ENSRNOG00000002541	574.8669315	0.264480904	0.07216727	0.013127443
Nrxn1	ENSRNOG00000050220	3921.497795	0.265939366	0.066612646	0.005488969
Tgfb2	ENSRNOG00000002418	536.048234	0.266703566	0.066378235	0.005247141
Trim44	ENSRNOG00000005191	959.198931	0.266774658	0.0825856	0.038257457
Pcsk6	ENSRNOG00000011526	458.3423759	0.267611501	0.06407337	0.003693286

Gene symbol	Ensembl gene	baseMean	log2FoldChange	lfcSE	*Padj*
Ppp3ca	ENSRNOG00000009882	3205.900141	0.2705459	0.055137378	0.000312481
Fer	ENSRNOG00000015898	242.7615793	0.270795374	0.081275659	0.030650724
Hspa4l	ENSRNOG00000010819	3437.179025	0.271087791	0.08308356	0.035606759
Pdpk1	ENSRNOG00000006136	614.9105463	0.271921311	0.073681963	0.012637868
Chm	ENSRNOG00000000161	628.4297981	0.27244317	0.085529519	0.042092259
Celf2	ENSRNOG00000023661	624.273415	0.27423324	0.081198515	0.028304517
Cdr2	ENSRNOG00000017260	389.1826626	0.274450707	0.081627761	0.02886135
Sptbn1	ENSRNOG00000005434	19121.17448	0.276612177	0.067258841	0.004180374
Tenm3	ENSRNOG00000012802	1507.201991	0.276979066	0.072363026	0.009019671
Itgav	ENSRNOG00000004912	634.4254214	0.27752495	0.089248232	0.048902275
Mdm4	ENSRNOG00000009696	417.8167975	0.277735338	0.07553697	0.012822556
Sacs	ENSRNOG00000014509	2604.299098	0.278776741	0.064213163	0.002309925
Bhlhe40	ENSRNOG00000007152	844.6807028	0.279544793	0.085622176	0.035541389
Epb41	ENSRNOG00000010037	315.7580891	0.279696457	0.080080572	0.02125918
Cux2	ENSRNOG00000001259	645.099898	0.279939979	0.073589355	0.00947744
Edil3	ENSRNOG00000033064	6779.093606	0.280109536	0.082955198	0.028304517
Trps1	ENSRNOG00000024998	307.7646326	0.281612375	0.072221523	0.007162916
Cndp2	ENSRNOG00000015591	1227.295408	0.282919997	0.089004731	0.042276382
Iws1	ENSRNOG00000014630	281.190001	0.284541227	0.071027982	0.00537028
Strbp	ENSRNOG00000010150	1535.856033	0.284804955	0.075736326	0.010362983
Otud7a	ENSRNOG00000015503	242.7584777	0.284837418	0.076414078	0.011347981
Mapk8	ENSRNOG00000020155	508.8276232	0.286718421	0.076070964	0.010215569
Akt3	ENSRNOG00000021497	1024.357922	0.287243796	0.068211751	0.003462609
Samd12	ENSRNOG00000043390	657.0791776	0.288272022	0.076411863	0.010207889
Htr2c	ENSRNOG00000030877	2622.351597	0.289250657	0.09093778	0.042230992
Apc	ENSRNOG00000020423	4102.384367	0.289630992	0.079890921	0.014726133
Xrn1	ENSRNOG00000042951	272.6105363	0.291271949	0.087120655	0.029752249
Gdap1	ENSRNOG00000005850	1914.275784	0.292126309	0.06985964	0.003693286
Robo1	ENSRNOG00000029614	760.1641278	0.293333382	0.090272409	0.036412954
Usp31	ENSRNOG00000025793	1000.931292	0.294043552	0.078395169	0.010597537
Bdp1	ENSRNOG00000017864	437.4333128	0.294613812	0.086141916	0.025435284
Sgpp2	ENSRNOG00000037695	1169.719569	0.295791363	0.087112803	0.027172193
Rbfox1	ENSRNOG00000002827	3516.582588	0.297521831	0.071812255	0.003916859
Igf1r	ENSRNOG00000014187	219.7394128	0.298661962	0.089826375	0.031125347
Insyn1	ENSRNOG00000026238	1490.857867	0.300542282	0.073805318	0.00466209
Ell2	ENSRNOG00000027089	419.8433158	0.302218571	0.095103326	0.042276382
Nfic	ENSRNOG00000004505	345.0894243	0.302219259	0.090730908	0.03067367
Dpf1	ENSRNOG00000020687	298.9619003	0.302369094	0.085439123	0.018933426
Fgd6	ENSRNOG00000054515	194.9808841	0.302472079	0.094736988	0.041338328
Tanc1	ENSRNOG00000025394	1540.349336	0.303867696	0.090813531	0.029752249
Palm2	ENSRNOG00000025558	235.5185325	0.304026517	0.095684994	0.042276382
Pcdh7	ENSRNOG00000012367	591.7743419	0.304103475	0.073451358	0.003929045
Zfp148	ENSRNOG00000001789	768.7611833	0.304911376	0.073486255	0.003869968
Elmo1	ENSRNOG00000059705	1777.153803	0.306854689	0.088282901	0.022441416

Gene symbol	Ensembl gene	baseMean	log2FoldChange	lfcSE	*Padj*
Zyg11b	ENSRNOG00000010859	803.8174165	0.308032526	0.096117102	0.040713898
Purb	ENSRNOG00000056150	1125.913794	0.310766734	0.076689924	0.004891246
Erc2	ENSRNOG00000015148	254.6216861	0.311006247	0.083132439	0.010882604
Klf9	ENSRNOG00000014215	1158.819887	0.31159501	0.072785634	0.002762785
Hspa12a	ENSRNOG00000018019	5988.32322	0.31176716	0.097364284	0.040732069
Lmbrd2	ENSRNOG00000054751	502.2481525	0.312434655	0.092752836	0.028632837
Rgs17	ENSRNOG00000018690	832.6681176	0.313908404	0.078909198	0.005667845
Nufip2	ENSRNOG00000024964	328.0383262	0.315051406	0.082545988	0.009298556
Gabra1	ENSRNOG00000003512	9069.897416	0.317241261	0.072317018	0.002009391
Phip	ENSRNOG00000008652	327.8827614	0.317811307	0.095074678	0.029752249
Pcdh9	ENSRNOG00000038068	1555.670793	0.317915267	0.077350479	0.004195403
Nudt4	ENSRNOG00000009094	4210.150761	0.320951061	0.102086648	0.045435563
Heg1	ENSRNOG00000001793	955.1132825	0.321700137	0.091142152	0.019308602
Cachd1	ENSRNOG00000010514	697.9434661	0.321742772	0.069318048	0.000933639
Peli2	ENSRNOG00000012363	209.1402376	0.322189338	0.093277744	0.023492971
Gabbr2	ENSRNOG00000008431	7424.934158	0.322954066	0.100354069	0.039248759
Kcna2	ENSRNOG00000018285	5446.869358	0.322995266	0.078132392	0.003981137
Nrg3	ENSRNOG00000033894	260.6934466	0.323330432	0.099159682	0.035695693
Samd9	ENSRNOG00000052444	498.203968	0.323774325	0.100011411	0.03747517
Atp2b2	ENSRNOG00000030269	7329.563909	0.324650217	0.079225649	0.004361739
Ppp1r9a	ENSRNOG00000008869	993.323359	0.326817205	0.065073596	0.000189563
Tcp11l1	ENSRNOG00000042576	253.6724866	0.329255473	0.097834783	0.028847937
Nek7	ENSRNOG00000000657	726.4878322	0.332250818	0.085025823	0.007014693
Lamb1	ENSRNOG00000005678	466.354878	0.334726007	0.093185755	0.016357655
Rc3h1	ENSRNOG00000002750	410.6404687	0.33500441	0.094734162	0.01907056
Cadm2	ENSRNOG00000030840	799.1587539	0.335314561	0.085736876	0.007014693
Atxn1	ENSRNOG00000016998	445.7298963	0.335978773	0.106962495	0.045526267
Plcd4	ENSRNOG00000016361	906.7425551	0.337678244	0.070988986	0.000596128
Cep350	ENSRNOG00000003882	682.1968287	0.338648247	0.082039318	0.004027128
Ro60	ENSRNOG00000003434	359.2794473	0.339846761	0.090187884	0.010215569
Cdyl	ENSRNOG00000032215	167.5713717	0.34183705	0.09735082	0.020242038
Adam22	ENSRNOG00000042478	616.3721878	0.342437277	0.083370613	0.004213755
Mib1	ENSRNOG00000013281	848.8008623	0.345465466	0.06137335	1.58E-05
Samd5	ENSRNOG00000023549	342.3108429	0.348054726	0.111482633	0.047706572
Setd7	ENSRNOG00000013045	879.2626664	0.348758429	0.080280811	0.002305952
Rasal2	ENSRNOG00000004917	445.6634117	0.349099314	0.091848342	0.00954818
Rsf1	ENSRNOG00000024194	421.3819128	0.35019465	0.105879302	0.032890127
Rgs8	ENSRNOG00000002369	3854.145232	0.350728803	0.094257381	0.01153139
Cox7b	ENSRNOG00000054689	244.210791	0.351200276	0.107525094	0.035492606
Ppard	ENSRNOG00000000503	439.1965597	0.351692429	0.102524434	0.024935242
Kcnip4	ENSRNOG00000032350	1839.619925	0.353171758	0.096503487	0.013297315
Nefh	ENSRNOG00000008716	3894.616023	0.353240463	0.111367162	0.042746541
Asxl2	ENSRNOG00000011908	240.5582581	0.353242747	0.106077045	0.030697759
Grm4	ENSRNOG00000000487	984.5874863	0.353815458	0.107066662	0.032994836
Crtac1	ENSRNOG00000015220	286.7697061	0.354608971	0.112314454	0.044139831

Gene symbol	Ensembl gene	baseMean	log2FoldChange	lfcSE	*Padj*
Mef2a	ENSRNOG00000047756	1940.120852	0.35543231	0.096494025	0.012700884
Cacna1e	ENSRNOG00000002863	367.7721656	0.356337191	0.112966682	0.044313506
Sgip1	ENSRNOG00000006357	724.8210396	0.356983642	0.085950117	0.003850694
Il1rap	ENSRNOG00000001928	539.5595443	0.35736474	0.108624638	0.033777464
Scn1a	ENSRNOG00000053122	4631.681763	0.357709625	0.09229085	0.007732659
Plcb4	ENSRNOG00000033119	3924.431117	0.359207715	0.108008567	0.03110396
Scn8a	ENSRNOG00000005309	2325.251685	0.359485967	0.077731232	0.000977133
Kcnc3	ENSRNOG00000019959	1930.434959	0.360994395	0.10548763	0.025398938
Epha4	ENSRNOG00000013213	3699.461648	0.361535703	0.112448595	0.039513521
Pex5l	ENSRNOG00000011211	1279.680465	0.363994938	0.101646618	0.016775071
Rab3ip	ENSRNOG00000005362	345.4333643	0.364043741	0.092824725	0.006831726
Sv2b	ENSRNOG00000011160	3094.292113	0.364183761	0.109970788	0.032482369
Btbd8	ENSRNOG00000023446	801.7773774	0.364417563	0.099175273	0.012822556
Tes	ENSRNOG00000051952	218.7614549	0.367496799	0.097562837	0.010231765
Sh3d19	ENSRNOG00000011752	1158.712004	0.367877817	0.098954424	0.011573321
Atp11c	ENSRNOG00000003472	165.61543	0.367944469	0.098682668	0.011347426
Chrnb2	ENSRNOG00000020778	1290.528567	0.369646675	0.097107024	0.00947744
Pde4d	ENSRNOG00000042536	513.1210067	0.372486005	0.087858503	0.003153558
Etl4	ENSRNOG00000008666	1477.450313	0.372862463	0.089368236	0.003693286
Rictor	ENSRNOG00000011341	476.2863086	0.373950394	0.081633404	0.001146069
Ano3	ENSRNOG00000004731	239.0090127	0.374919452	0.099354368	0.010207889
Ppm1e	ENSRNOG00000024730	1442.624307	0.379661862	0.091734462	0.003929045
Clmn	ENSRNOG00000011044	1593.385213	0.380740298	0.088181415	0.002517438
Grm1	ENSRNOG00000014290	1908.226329	0.381674115	0.082694211	0.001004031
Rif1	ENSRNOG00000054901	236.5691281	0.382329823	0.086495483	0.001763926
Cdkn1a	ENSRNOG00000000521	202.0414899	0.383271121	0.116987173	0.03479799
Nrn1	ENSRNOG00000050767	1659.179531	0.386294531	0.107736838	0.01659457
Unc80	ENSRNOG00000028362	2261.779329	0.389133869	0.082918345	0.00075438
Syt7	ENSRNOG00000026432	2119.969868	0.390277753	0.119645269	0.035639477
Ift57	ENSRNOG00000001958	613.95574	0.390503964	0.08932147	0.002068605
Scrt1	ENSRNOG00000025594	777.9504267	0.391220833	0.105446669	0.011830826
Megf9	ENSRNOG00000005932	1573.563718	0.391607365	0.104509512	0.010711179
Zmat4	ENSRNOG00000042690	1499.615887	0.396895331	0.11589604	0.025327589
Mcf2	ENSRNOG00000003435	237.7530469	0.400127659	0.127333533	0.045485769
Cntn4	ENSRNOG00000005652	981.8459078	0.400743847	0.09343769	0.002753066
Kcnj6	ENSRNOG00000001658	331.5172766	0.401343131	0.117298069	0.025398938
Peak1	ENSRNOG00000042519	761.0209982	0.402043206	0.099091776	0.004850082
Nhlh2	ENSRNOG00000054375	201.0106531	0.403549401	0.119925631	0.028847937
Plekhd1	ENSRNOG00000038297	536.9216184	0.403916263	0.113785047	0.018465299
Ntng1	ENSRNOG00000031136	3270.248212	0.404475414	0.126268307	0.040732069
Ttll7	ENSRNOG00000031997	3318.861945	0.405211368	0.098164368	0.004027128
Nefm	ENSRNOG00000013916	4552.365785	0.410015299	0.097837638	0.003653197
Fam135b	ENSRNOG00000005159	170.0353135	0.410423992	0.125951001	0.035830642
Hecw1	ENSRNOG00000016046	533.4493791	0.412579485	0.068915961	4.54E-06

Gene symbol	Ensembl gene	baseMean	log2FoldChange	lfcSE	*Padj*
Hecw2	ENSRNOG00000013257	505.2668652	0.413402839	0.103204596	0.00537028
Etv1	ENSRNOG00000006867	1777.174967	0.413410543	0.080802717	0.00014459
Prex2	ENSRNOG00000005391	1512.83793	0.413418351	0.069849778	6.02E-06
Reps2	ENSRNOG00000061508	354.9253593	0.417526032	0.120921686	0.023532286
Vav3	ENSRNOG00000020485	875.9139404	0.418182077	0.130534965	0.040732069
Itpr1	ENSRNOG00000007104	3867.757846	0.418275324	0.127003048	0.033777464
Inpp4b	ENSRNOG00000018382	527.9499988	0.420801362	0.129221731	0.036028218
Nhsl2	ENSRNOG00000037951	236.1659712	0.423771752	0.093482702	0.001250515
Cacnb4	ENSRNOG00000007666	1420.294682	0.424123592	0.115955256	0.013308656
Psd3	ENSRNOG00000013884	687.2806127	0.425256593	0.094234571	0.001301707
Slc1a2	ENSRNOG00000005479	2753.51477	0.425332564	0.083378985	0.000151871
Wnt4	ENSRNOG00000013166	461.3861389	0.426867681	0.136343812	0.046555372
Pclo	ENSRNOG00000005726	1972.262608	0.427973792	0.09761718	0.002009964
Pde7b	ENSRNOG00000013436	350.8623225	0.427979384	0.120599566	0.018479795
Frrs1l	ENSRNOG00000043103	324.0531853	0.428668937	0.089303	0.000511763
Tpd52l1	ENSRNOG00000021478	587.2757739	0.429752093	0.114015899	0.010215569
Deptor	ENSRNOG00000004328	279.4014482	0.430714291	0.106563585	0.005004494
Homer2	ENSRNOG00000061450	238.3419818	0.433060261	0.127320999	0.02676801
Atrnl1	ENSRNOG00000017406	1205.623621	0.437396471	0.124555109	0.020242038
Slc8a1	ENSRNOG00000008479	575.5018949	0.437912577	0.091968806	0.000594353
Adarb2	ENSRNOG00000030775	296.9756259	0.439262273	0.130291088	0.028548818
Rorb	ENSRNOG00000013413	309.6686772	0.442337329	0.096908689	0.001175179
Stxbp5l	ENSRNOG00000002496	216.4718376	0.443456449	0.103459694	0.002753066
Cdh6	ENSRNOG00000013535	337.8615289	0.448985937	0.107648087	0.003693286
Fat3	ENSRNOG00000011585	504.3183016	0.449467024	0.098309653	0.001157267
Osbpl3	ENSRNOG00000010011	527.7394698	0.450116922	0.142562767	0.044139831
Plekhg1	ENSRNOG00000016011	3339.080658	0.453574452	0.130567502	0.022471076
Slitrk6	ENSRNOG00000022337	1020.405141	0.454370983	0.109429826	0.003850694
Kcnma1	ENSRNOG00000005985	543.2732598	0.45470467	0.11952827	0.00947744
Birc6	ENSRNOG00000027191	1025.24224	0.458729193	0.090987001	0.000180303
Plcxd2	ENSRNOG00000042289	684.4730097	0.460525856	0.098781286	0.00086079
Pdk3	ENSRNOG00000012513	302.4202675	0.464603249	0.092670838	0.000192035
Rasgrp1	ENSRNOG00000005404	2467.037729	0.46564293	0.13724672	0.027204931
Sprn	ENSRNOG00000018927	207.317064	0.465748739	0.136600142	0.026111624
Vps13c	ENSRNOG00000030213	950.0397114	0.466069531	0.074953671	1.87E-06
Tcf7l2	ENSRNOG00000049232	4399.174822	0.46724448	0.119028805	0.006800616
Bmpr2	ENSRNOG00000022196	762.7642699	0.469337267	0.114123306	0.004180374
Megf11	ENSRNOG00000010634	174.4524558	0.472406178	0.124533888	0.009648773
Igf1	ENSRNOG00000004517	108.2188893	0.474015573	0.140382403	0.028304517
Myo9a	ENSRNOG00000011619	820.8794678	0.476125558	0.09420641	0.000178394
Usf3	ENSRNOG00000027756	345.9507072	0.476975492	0.093612352	0.000152131
Lrrc7	ENSRNOG00000011980	603.3196208	0.477879029	0.141867846	0.028632837
Camk1d	ENSRNOG00000017882	939.5332303	0.478390526	0.095013089	0.000181942
Tcf7l2	ENSRNOG00000013993	255.862825	0.484932063	0.14377766	0.028548818
Kcna1	ENSRNOG00000019750	775.3571592	0.486624594	0.129673823	0.010597537

Gene symbol	Ensembl gene	baseMean	log2FoldChange	lfcSE	*Padj*
Grin2b	ENSRNOG00000008766	422.086955	0.489294239	0.11778057	0.003850694
Ttbk2	ENSRNOG00000011059	720.0809041	0.497504657	0.08785048	1.54E-05
Atp2b1	ENSRNOG00000004026	7693.592131	0.498620983	0.128910246	0.007910703
Orai1	ENSRNOG00000001336	81.81265456	0.499461949	0.145884351	0.025341076
Tmem245	ENSRNOG00000026271	199.2472763	0.500209251	0.110236714	0.001242515
Vps13a	ENSRNOG00000025539	404.007429	0.501713887	0.12187628	0.004180374
Nwd2	ENSRNOG00000051837	379.1613124	0.501754456	0.145513386	0.023877063
Pdp1	ENSRNOG00000016180	2728.034995	0.505123266	0.135790295	0.01153139
Csrnp3	ENSRNOG00000005359	148.4593358	0.511736709	0.128312022	0.005522386
Cck	ENSRNOG00000019321	1667.652576	0.513605844	0.130123319	0.00635003
Lef1	ENSRNOG00000010121	1046.838232	0.51410067	0.139546385	0.012700884
Hdac9	ENSRNOG00000004158	322.5462952	0.514944837	0.120082043	0.002753066
Galnt14	ENSRNOG00000007951	189.6676771	0.516846751	0.120146659	0.002648079
Gabrb2	ENSRNOG00000003680	1379.920674	0.518466231	0.121874695	0.003055669
Wnt9b	ENSRNOG00000003807	646.7922202	0.520452206	0.151667532	0.024935242
Pcp4	ENSRNOG00000001628	7267.736506	0.521930955	0.129100524	0.005004494
Slc24a2	ENSRNOG00000008169	7828.314658	0.523062091	0.115524652	0.001264847
Gpr63	ENSRNOG00000007675	363.3619141	0.527608211	0.161504515	0.035492606
Plvap	ENSRNOG00000017676	116.4566626	0.533028099	0.145209476	0.012917407
Hs6st3	ENSRNOG00000037886	172.6488075	0.533687765	0.131605909	0.004861569
Synpo2	ENSRNOG00000014867	1770.094501	0.534840395	0.164297648	0.03609236
Wipf3	ENSRNOG00000009571	889.3737813	0.551046079	0.176114077	0.046773739
Car7	ENSRNOG00000012371	159.0435427	0.551865653	0.135601226	0.00466209
Hlf	ENSRNOG00000002456	555.5321977	0.551893202	0.096495875	1.44E-05
Arfgef3	ENSRNOG00000011460	573.8296221	0.563182783	0.109560375	0.000137611
Acvr1c	ENSRNOG00000004828	128.5733288	0.565763011	0.167858955	0.028571374
Epha6	ENSRNOG00000029184	118.9208572	0.569092572	0.177992442	0.040928336
Il1rapl1	ENSRNOG00000029663	66.77481072	0.581345489	0.173639494	0.029731932
Myo5a	ENSRNOG00000058866	4283.3543	0.584020053	0.096730521	3.87E-06
Rab3c	ENSRNOG00000011623	1603.495121	0.595603448	0.111972881	6.73E-05
Dok6	ENSRNOG00000038190	205.0391255	0.596017961	0.113608992	9.22E-05
Lypd6	ENSRNOG00000038980	486.0666468	0.605374767	0.128647659	0.00072252
Prkcq	ENSRNOG00000019057	532.9662267	0.605647912	0.11917238	0.000158355
Taok1	ENSRNOG00000015692	476.7977728	0.606365201	0.121021285	0.000192035
Agtr2	ENSRNOG00000050006	82.11303132	0.611837857	0.192232976	0.042150762
Gabrb1	ENSRNOG00000002327	248.1106806	0.615714304	0.138551475	0.001601065
Lyst	ENSRNOG00000058094	294.475479	0.618237689	0.111526173	2.32E-05
Lhx9	ENSRNOG00000010357	119.7428395	0.634213901	0.186944932	0.027204931
Sema3d	ENSRNOG00000007202	277.5519749	0.63556307	0.133147002	0.000572309
Ogfrl1	ENSRNOG00000014142	672.9820351	0.644447117	0.116040218	2.31E-05
Map3k2	ENSRNOG00000014089	54.37080402	0.651791018	0.168819777	0.007989083
Rgs7bp	ENSRNOG00000013389	462.9928361	0.653139704	0.145218329	0.001360407
Wnk3	ENSRNOG00000002537	224.3004118	0.655906943	0.125457189	9.42E-05
Kdm7a	ENSRNOG00000052445	131.0223207	0.659558717	0.152104999	0.002339728

Gene symbol	Ensembl gene	baseMean	log2FoldChang	lfcSE	*Padj*
Rpl17	ENSRNOG00000018680	138.6038545	0.665491418	0.207586254	0.040642152
Foxp2	ENSRNOG00000054508	803.8614382	0.668070433	0.108850728	2.49E-06
Rab37	ENSRNOG00000059793	208.9671674	0.668275399	0.152704579	0.00206062
Tet1	ENSRNOG00000000277	56.18163159	0.671871663	0.175840165	0.00918244
Mdfic	ENSRNOG00000053787	92.34536377	0.673614752	0.175590028	0.008748688
Cnih3	ENSRNOG00000022724	53.74365365	0.674533638	0.188716873	0.017095599
Nmbr	ENSRNOG00000012103	68.22109562	0.702858367	0.188843779	0.01153139
Ago3	ENSRNOG00000034269	56.4487641	0.711830451	0.177919734	0.005372388
Ptar1	ENSRNOG00000014891	68.35082952	0.716784175	0.158816045	0.001301707
Camk4	ENSRNOG00000020478	208.2828586	0.725505177	0.126297767	1.37E-05
Cdkl5	ENSRNOG00000003742	557.9017041	0.748865636	0.185322223	0.005004494
Kcnq3	ENSRNOG00000005206	400.2331286	0.753916802	0.140994763	6.03E-05
Cbln4	ENSRNOG00000004372	882.2620517	0.777149684	0.183359179	0.003153558
Xkr4	ENSRNOG00000027276	92.38978755	0.777160921	0.170457687	0.001175179
Scai	ENSRNOG00000025278	53.48191385	0.809827579	0.180883196	0.001440596
Fzd3	ENSRNOG00000047211	228.7564792	0.85390625	0.15062574	1.54E-05
Lnpep	ENSRNOG00000055229	87.45976602	0.857664592	0.158633549	4.54E-05
Lypd6b	ENSRNOG00000004614	245.6907108	0.913778969	0.236407357	0.007959998
Fzd5	ENSRNOG00000014678	44.87895786	0.918936444	0.200930919	0.001157267
Ranbp2	ENSRNOG00000056428	47.31844126	0.920783573	0.188534641	0.00034321
Lox	ENSRNOG00000014426	193.8606449	0.941062001	0.199768962	0.000718589
Kcnh7	ENSRNOG00000007528	56.13176725	0.94600842	0.167619242	1.54E-05
Zfp871	ENSRNOG00000004947	92.79826377	0.974111601	0.155426135	1.82E-06
Tacstd2	ENSRNOG00000007740	19.8355427	0.987632493	0.273467505	0.015427318
Grin2a	ENSRNOG00000033942	294.2355249	1.015231009	0.151511534	1.54E-07
Lnpep	ENSRNOG00000047387	80.97569214	1.044381904	0.18130361	1.37E-05
Ctxn3	ENSRNOG00000022957	248.7995159	1.048900831	0.304409792	0.023939083
Acpp	ENSRNOG00000011820	30.04738876	1.115039909	0.277599184	0.005247141
Rxfp2	ENSRNOG00000000897	26.2506957	1.119101665	0.249628404	0.001423305
Rora	ENSRNOG00000027145	170.8501767	1.150319757	0.202007983	1.53E-05
Oprm1	ENSRNOG00000018191	187.0866108	1.291965669	0.244218088	7.56E-05
Fut9	ENSRNOG00000008475	46.37577146	1.390292912	0.270826901	0.000137611
Dgkh	ENSRNOG00000010065	26.99447935	1.519118089	0.276274654	2.84E-05
Kcnh5	ENSRNOG00000009542	82.70350855	1.656408202	0.199404056	1.46E-12
NA	ENSRNOG00000051264	29.76124212	-1.152153965	0.301478531	0.00918244
NA	ENSRNOG00000029586	39.22536675	-0.67701781	0.211640601	0.0408056
NA	ENSRNOG00000061046	624.7532131	0.209286314	0.059643383	0.020365793
NA	ENSRNOG00000022709	402.4343716	0.244808239	0.070300584	0.022034708
NA	ENSRNOG00000048800	293.4544258	0.256555039	0.08011618	0.040732069
NA	ENSRNOG00000054121	381.9155876	0.286056455	0.082341727	0.022471076
NA	ENSRNOG00000062140	103.9979935	0.407054762	0.117443276	0.023073775
NA	ENSRNOG00000057161	200.122593	0.407578651	0.131211523	0.04897967
NA	ENSRNOG00000030330	158.1770424	0.41161199	0.13203228	0.048258418
NA	ENSRNOG00000054085	102.3189468	0.556533205	0.170475765	0.035541389
NA	ENSRNOG00000053968	179.7681396	0.743346979	0.142073502	9.42E-05

Gene symbol	Ensembl gene	baseMean	log2FoldChang(	lfcSE	*Padj*
NA	ENSRNOG00000060991	124.8955663	0.676460553	0.181007852	0.011008909
NA	ENSRNOG00000059746	38.1621653	1.182431669	0.230421591	0.000137611
NA	ENSRNOG00000058217	19.16365569	1.525121791	0.322459685	0.000667926
RGD1560108	ENSRNOG00000032042	259.2682018	-0.36965735	0.10168995	0.014324551
RGD1561327	ENSRNOG00000031222	703.4561693	-0.309345564	0.083265949	0.011643133
RGD1305110	ENSRNOG00000054669	792.6574318	0.190145133	0.059761414	0.042209922
RGD1311739	ENSRNOG00000021245	368.9821364	-0.249740131	0.076304165	0.03504142
RGD1307100	ENSRNOG00000038436	3835.515925	0.301653286	0.060440915	0.000207559
RGD1561481	ENSRNOG00000012086	130.6792645	0.309328129	0.098997774	0.047380799
RGD1306271	ENSRNOG00000013729	885.2716318	0.365795747	0.082209666	0.001597228
LOC1025529	ENSRNOG00000027022	704.9354277	-0.281166876	0.082030716	0.025118976
LOC1083482	ENSRNOG00000020062	64.86580183	0.492186562	0.150006368	0.034462588
LOC1009098	ENSRNOG00000045593	27.42880398	0.745999153	0.210098285	0.018462067

Supplemental Table S2. Transcription factor enrichment analysis performed in ChIP-seq Enrichment Analysis tool, v. 3 (ChEA3). The top 10 ranking transcription factors from the integrated mean rank libraries in ChEA3 are reported. Transcription factor enrichment analysis results are provided for the entire control-RS7 comparison gene list (A), for the up-regulated gene list from the control-RS7 comparison (B) and for the down-regulated gene list from the control-RS7 comparison (C). A similar transcription factor enrichment analysis is provided for the entire AS-RS7 comparison gene list (D), for the up-regulated gene list from the AS-RS7 comparison (E) and for the down-regulated gene list from the AS-RS7 comparison (F).

A. Transcription factor enrichment analysis on entire control-RS7 comparison gene list.

Rank	Transcr. Factor	Mean Rank	Overlapping Genes
1	Myt1l	11.0	73
2	Zmat4	13.0	69
3	Znf365	13.0	78
4	Camta1	20.67	67
5	Csrnp3	21.0	54
6	Znf385b	25.33	67
7	Peg3	26.5	63
8	Znf804a	27.0	71
9	Zbtb18	28.5	47
10	Dach2	29.0	57

B. Transcription factor enrichment analysis on upregulated control-RS7 comparison gene list.

Rank	Transcr. Factor	Mean Rank	Overlapping Genes
1	Myt1l	8.0	64
2	Znf365	9.33	69
3	Camta1	15.33	60
4	Znf385b	16.67	60
5	Zmat4	17.0	58
6	Znf804a	24.33	59
7	Csrnp3	26.0	48
8	Purg	29.5	37
9	Dach2	30.33	47
10	Zbtb18	32.0	42

C. Transcription factor enrichment analysis on downregulated control-RS7 comparison gene list.

Rank	Transcr. Factor	Mean Rank	Overlapping Genes
1	Dlx6	9.33	17
2	Lhx8	10.33	18
3	Sp9	22.0	9
4	Sox14	32.0	19
5	Six6	33.0	21
6	Otp	35.0	18
7	Meis2	36.75	25
8	Dlx1	40.0	14
9	Pou3f4	42.67	21
10	Arx	48.0	16

D. Transcription factor enrichment analysis on entire AS-RS7 comparison gene list.

Rank	Transcr. Factor	Mean Rank	Overlapping Genes
1	Csrnp3	8.0	80
2	Peg3	8.5	94
3	Myt1l	9.0	107
4	Zmat4	12.67	111
5	Zbtb41	16.67	105
6	Dach2	18.33	90
7	Ash1l	20.33	90
8	Rorb	21.0	113
9	Znf385b	25.67	95
10	Zbed6	27.0	42

E. Transcription factor enrichment analysis on upregulated AS-RS7 comparison gene list.

Rank	Transcr. Factor	Mean Rank	Overlapping Genes
1	Myt1l	6.67	97
2	Ash1l	11.0	89
3	Zbtb41	15.33	99
4	Zmat4	17.33	97
5	Zbed6	20.0	42
6	Csrnp3	22.5	69
7	Rorb	27.33	101
8	Znf385b	29.0	84
9	Dach2	29.33	72
10	Znf510	30.0	89

F. Transcription factor enrichment analysis on downregulated AS-RS7 comparison gene list.

Rank	Transcr. Factor	Mean Rank	Overlapping Genes
1	Dlx1	28.67	22
2	Lhx3	31.67	30
3	Sgsm2	33.0	17
4	Dlx2	48.0	19
5	Dlx6	48.0	17
6	Sp9	51.0	13
7	Foxg1	55.33	19
8	Lhx8	68.33	18
9	Dmbx1	68.33	23
10	Insm2	71.33	18
